# Design, Synthesis, Antimicrobial Activity, and Molecular Docking of Novel Thiazoles, Pyrazoles, 1,3-Thiazepinones, and 1,2,4-Triazolopyrimidines Derived from Quinoline-Pyrido[2,3-*d*] Pyrimidinones

**DOI:** 10.3390/ph17121632

**Published:** 2024-12-04

**Authors:** Ameen Ali Abu-Hashem, Sami A. Al-Hussain

**Affiliations:** 1 Department of Physical Sciences, Chemistry Division, College of Science, Jazan University, Jazan 45142, Saudi Arabia; 2 Department of Chemistry, Faculty of Science, Imam Mohammad Ibn Saud Islamic University (IMSIU), Riyadh 11623, Saudi Arabia; sahussain@imamu.edu.sa

**Keywords:** pyrido[2,3-*d*] pyrimidine, quinoline, pyridothiazolopyrimidinone, 1,2,4-triazolopyrimidine, thiazole, pyrazole, hydrazine, 1,3-thiazepinone, molecular docking, antimicrobial activity

## Abstract

Background: Recently, pyrido[2,3-*d*] pyrimidine, triazolopyrimidine, thiazolopyrimidine, quinoline, and pyrazole derivatives have gained attention due to their diverse biological activities, including antimicrobial, antioxidant, antitubercular, antitumor, anti-inflammatory, and antiviral effects. Objective: The synthesis of new heterocyclic compounds including 5-quinoline-pyrido[2,3-*d*] pyrimidinone (**1**–**2**, **4**, **6**–**7**), 6-quinoline-pyrido[2,3-*d*]thiazolo[3,2-*a*]pyrimidinone (**3**, **5**, **8**–**10**), 1,2,4-triazole-6-quinoline-pyrido[2,3-*d*]thiazolo[3,2-*a*]pyrimidinone (**11**–**13**), and pyrido[2,3-*d*]thiazolo[3,2-*a*]pyrimidine-ethyl-(pyridine)-9-thiaazabenzo[*cd*]azulenone (**14**) derivatives was performed with high yields while evaluating antimicrobial activities. Methods: A new series of quinoline-pyrido[2,3-*d*]thiazolo[3,2-*a*]pyrimidine derivatives were prepared using a modern style and advanced technology, resulting in high yields of these new compounds. Various reagents were utilized, specifically tailored to the production needs of each compound, through reactions that included alkylation, addition, condensation, acylation, the formation of Schiff bases, and intramolecular cyclization. Results: The chemical structures of the new compounds were determined using spectroscopy analyses, including IR, NMR, and MS, achieving good yields ranging from 68% to 90% under mild conditions in a regular system. All compounds were tested for in vitro antimicrobial activity and compared to standard drugs, specifically cefotaxime sodium and nystatin. The results showed that compounds **10** to **14** exhibited excellent antimicrobial activity, with a minimum inhibitory concentration (MIC) of 1 to 5 µmol/mL, compared to that of the standard drugs, which had MIC values of 1 to 3 µmol/mL. Furthermore, molecular docking studies were conducted to explore the interactions of specific compounds with antimicrobial target proteins. The findings revealed that compounds **10** to **14** displayed significant binding energies, with ΔG values ranging from −7.20 to −11.70 kcal/mol, indicating effective binding to the active sites of antimicrobial protein receptors. Conclusions: The SAR study confirmed a relationship between antimicrobial activity and the tested compounds. Molecular docking demonstrated that compounds **10**, **11**, **12**, **13**, and **14** exhibited significant binding energy, effectively interacting with the active sites of antimicrobial protein receptors. This consistent finding supports that these new compounds’ practical and theoretical studies align regarding their antimicrobial activity.

## 1. Introduction

**Figure 1:**
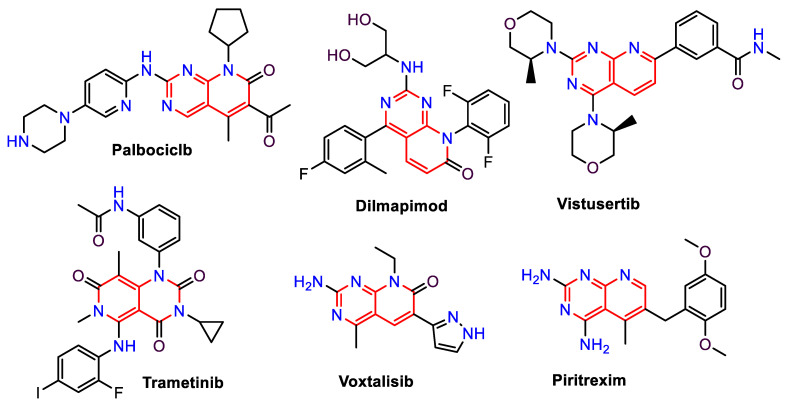
Some drugs with interesting molecules of Pyrido[2,3-*d*] pyrimidine derivatives (all of these drugs contain the Pyrido[2,3-*d*] pyrimidine moiety, so this moiety is red, and all the nitrogen atoms are blue.).

The nitrogen atom is found in pyridine and pyrimidine, forming *N*-heterocycles. Many possible structures combine pyrimidine and pyridine rings. Pyridopyrimidines and similar compounds are of great interest due to their biological activity. The pyridopyrimidine structure is present in essential drugs and has been the subject of numerous publications, studies, and clinical trials in recent years, showing promise for developing new therapies [1,2,3]. Pyrido[2,3-*d*] pyrimidine derivatives are commonly found in biological molecules. For example, they are involved in dihydrofolate reductase (DHFR), tyrosine-protein kinase (Abl, MAP) kinases, and biotin-carboxylase. The pyridopyrimidine medicine inhibits dihydrofolate reductase (DHFR), which has a high affinity. This reduces the amount of tetrahydrofolate needed to produce pyrimidine and purine. As a result, the synthesis of RNA and DNA is halted, leading to the destruction of cancer cells [4,5]. Inhibitors of this enzyme are being researched and used to treat various diseases, such as rheumatoid arthritis and psoriasis [6,7]. The pyrido[2,3-*d*] pyrimidine moieties are considered one of the most important classes of heterocyclic compounds. They have various pharmacological and biological activities, including antioxidant [8,9,10,11], antimicrobial [12,13,14], antitubercular [15], anti-inflammatory [16,17,18], antiviral [19], adenosine kinase inhibitor [20], and efficient glucosidase inhibitor [21,22]. Our bibliographic investigation reveals the utilization of various pyrido[2,3-*d*] pyrimidine and its derivatives in numerous therapeutic applications. Among these drugs are **Palbociclib** (a cyclin-dependent kinase inhibitor for breast cancer), **Dilmapimod** (showing potential activity against rheumatoid arthritis), **Vistusertib** (the novel mTOR inhibitor with kinase activity), **Trametinib** (a dual-specificity mitogen-activated protein kinase inhibitor), **Voxtalisib** (a PI3K/mTOR inhibitor with kinase activity), and **Piritrexim** (a DHFR dihydrofolate reductase inhibitor) [23], as illustrated in Figure 1.

**Figure 2:**
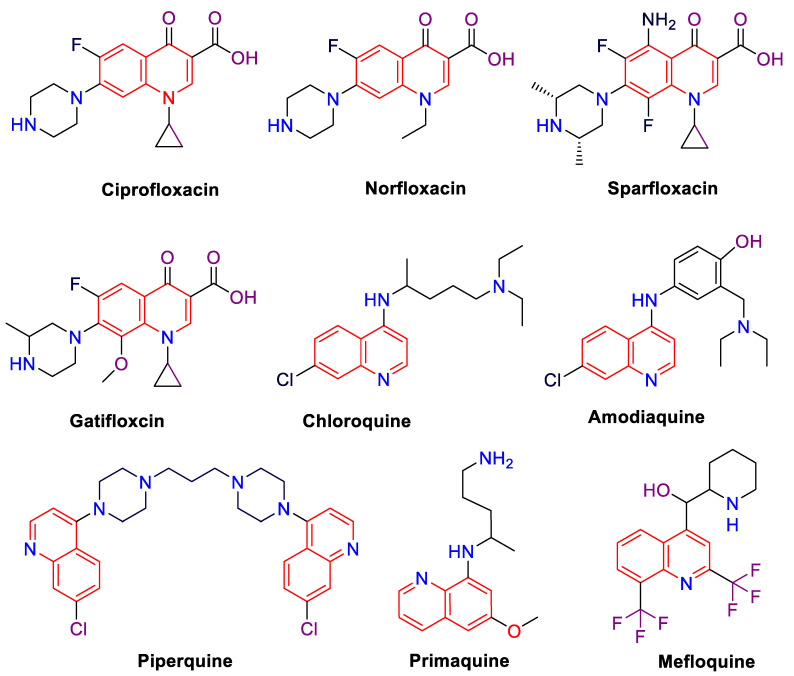
Certain drugs contain derivatives of quinoline (all of these drugs contain the quinoline moiety, so this moiety is red, and all the nitrogen atoms are blue).

Many natural compounds contain heterocyclic compounds, including those in the alkaloid family. The most famous compounds in this family are quinoline and its derivatives, which can be extracted from various natural sources such as the roots of *Zanthoxylum wutaiense* and contain quinoline alkaloids (**dictamnine** and **γ-fagarine**), which have demonstrated moderate antitubercular activity (against the H37Rv strain) [24]. In addition, (**Berberine**), an active compound extracted from *Berberis aristata*’s stem, has antimicrobial activity against a drug-resistant strain of (*Helicobacter pylori*, anti-*H. Pylori*) therapy [25]. Also, *Chelidonium majus* (**Papaveraceae**) extracts display antimicrobial activity due to their complex alkaloid composition. Moreover, these compounds (**chelerythrine** and **chelidonine**) have antifungal activity against *C. albicans* [26]. In addition, (**Sanguinarine**) is quinoline salt, a toxic polycyclic extracted from *Chelidonium majus*, *Macleaya cordata*, and *Argemone mexicana*. It kills animal cells by acting on the transmembrane protein (Na^+^/K^+^-ATPase) [27,28]. Due to the biological significance of quinoline and its derivatives, researchers have synthesized numerous quinoline derivatives with diverse biological activities, such as antioxidant [29], antiviral [30], antimicrobial [31,32], antimalarial [33], anti-inflammatory [34], antihypertensive [35], anti-asthmatic [36], analgesic [37], anticancer [38], and antileishmanial [39] ones. Also, quinolines, quinazolines, and 1,2,4-triazoles with pyrido[2,3-*d*] pyrimidines are utilized for their anticancer activities [40]. Quinoline is a component of several drugs used in clinical settings, particularly in antimalarial medications. It has also been utilized in medicines for other diseases, including fluoroquinolone antibiotics such as **Ciprofloxacin** and its analogues **Norfloxacin**, **Sparfloxacin**, and **Gatifloxacin** [41]. Aminoquinoline has been a critical component of antimalarial medications since the 1940s. The first drug in this class, **chloroquine**, was discovered in 1934 [42]. Due to resistance to **chloroquine**, a series of its analogues (e.g., **Amodiaquine**, **Piperaquine**, **Primaquine**, and **Mefloquine**) were developed [43]. The structures of these compounds are shown in Figure 2.

Many researchers have also studied and synthesized heterocyclic compounds such as pyrimidines, pyridines, quinolines, pyrazoles, triazoles, thiazoles, oxadiazoles, pyrroles, and others due to their biological significance [44,45,46,47,48,49], especially their antimicrobial activities [50,51,52,53,54,55,56,57,58,59,60,61,62,63,64,65,66,67,68,69,70].

Moreover, in our previous scientific studies, we synthesized numerous heterocyclic compounds and examined their biological activity [71,72,73,74,75,76,77,78,79,80,81,82]. In our research, we developed a new method for creating 3-substituted-phenyl-6-(quinoline)-pyrido[2,3-*d*]thiazolo[3,2-*a*] pyrimidinones. This process combines thiazole, pyrazole, and thioxo-pyrimidine with quinoline-pyrido[2,3-*d*] pyrimidinones. Also, it involves hydroxy-imino, 4-substituted (phenyl) acryloyl, and hydrazine-1-carbothio-amide to connect quinoline-pyrido[2,3-*d*]thiazolo[3,2-*a*] pyrimidines, in addition to 1,3-thiazepinone and 1,2,4-triazolopyrimidine derivatives. We conveniently used new reagents and techniques to achieve excellent yields for new organic products. These new compounds were evaluated in vitro for their antimicrobial activities against various bacteria and fungi.

## 2. Results and Discussion

### 2.1. Synthesis

**Scheme 1:**
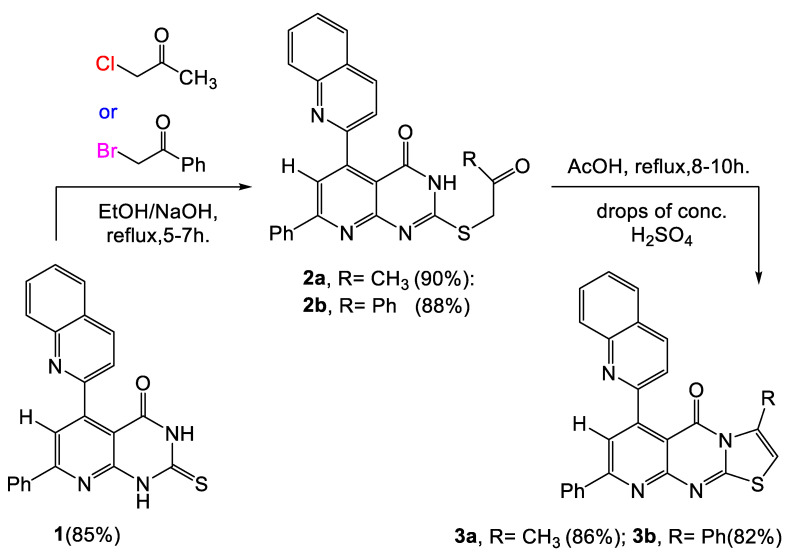
Synthesis of 3-sub-phenyl-6-(quinoline)-pyrido[2,3-*d*]thiazolo[3,2-*a*]pyrimidinones (to clarify the type of reagents, one contains chlorine, and the other contains bromine; therefore, they are represented in different colours).

The reaction of 7-phenyl-5-(quinolin-2-yl)-2-thioxo-2,3-dihydropyrido[2,3-*d*] pyrimidin-4(1*H*)-one (**1**) [32,40] in an ethanolic potassium hydroxide solution with α-haloketones such as chloroacetone and phenacyl bromide yielded 2-((2-oxo-substituted)thio)-7-phenyl-5-(quinolin-2-yl) pyrido[2,3-*d*]pyrimidin-4(3H)-one (**2a**, **b**). The assignment of structure (**2a**, **b**) to the reaction products was based on correct elemental analysis. Also, the IR spectra agreed with the structure and revealed the broad (NH) group ν 3250–3255 cm^−1^ and two carbonyl groups around ν 1690–1725 cm^−1^. The ^1^H-NMR spectrum of (**2a**) showed an observed singlet at δ 9.10 ppm, representing the existence of one proton of the (NH) group, which is exchangeable with (D_2_O). The latter compounds (**2a**, **b**) underwent cyclization when boiled with glacial acetic acid in the presence of the catalytic amount of sulfuric acid to give 3-substituted-8-phenyl-6-(quinolin-2-yl)-5H-pyrido[2,3-*d*]thiazolo[3,2-*a*] pyrimidin-5-one (**3a**, **b**). The structure of (**3a**, **b**) was preferred based on ^1^H-NMR and ^13^C-NMR spectral data. Thus, with the ^1^H-NMR spectrum of (**3a**), three singlets at δ 2.23, 4.90, and 8.01 ppm were observed, corresponding to three protons of methyl, one proton of thiazole, and one proton of the pyridine ring, respectively. Moreover, the ^13^C-NMR spectrum of (**3a**) had signals at δ 21.2 ppm for one carbon atom of the methyl group, 97.5 ppm for one carbon atom of the thiazole ring; 118.5 ppm for one carbon atom of the pyridine ring; and 119.1, 120.4, 121.7, 125.3, 127.2, 127.4, 127.8, 128.1, 129.3, 129.8, 138.2, 139.1, 141.1, 144.5, 150.4, 154.1, 155.3, 156.1, 158.5 (21C, Ar-C, sp^2^ carbon atoms), and 166.2 ppm for one carbonyl group. Also, the mass spectrum of (**3a**) showed a molecular ion peak at *m*/*z* 420 for [M^+^], (100%), as shown in Scheme 1.

**Scheme 2:**
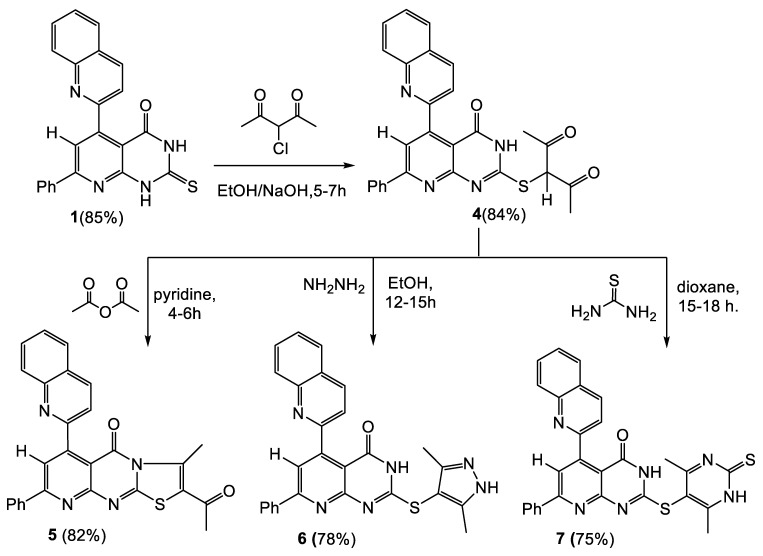
Synthesis of thiazole, pyrazole, and thioxopyrimidine with quinoline-pyrido[2,3-*d*] pyrimidinone derivatives.

The alkylation of an ethanolic sodium hydroxide solution of 7-phenyl-5-(quinolin-2-yl)-2-thioxo-2,3-dihydropyrido[2,3-*d*] pyrimidin-4(1*H*)-one (**1**) with 3-chloro-2,4-pentane-dione yielded 3-((4-oxo-7-phenyl-5-(quinolin-2-yl)-3,4-dihydropyrido[2,3-*d*] pyrimidin-2-yl)thio) pentane-2,4-dione (**4**). Compound (**4**) was investigated using IR and ^1^H NMR spectroscopy. The IR spectrum displayed absorption bands at *ν* 3270 cm^−1^, representing the existence of the (NH) group, and at 1724, 1719, and 1681 cm^−1^, confirming the presence of three carbonyl groups. The ^1^H-NMR spectra showed the presence of one singlet proton at *δ* 8.01 ppm of the pyridine ring, with one proton appearing as a singlet at *δ* 10.68 ppm of the (NH) group exchangeable with D_2_O. The reactions of 2-alkylthio (**4**) are a significant area of study in organic chemistry, as they can form different products depending on the reagents and conditions used. For instance, when compound (**4**) was boiled with a mixture of acetic anhydride-pyridine (20 mL, 1:1), it underwent cyclization to produce 2-acetyl-3-methyl-8-phenyl-6-(quinolin-2-yl)-5*H*-pyrido[2,3-*d*]thiazolo[3,2-*a*] pyrimidin-5-one (**5**). The IR spectrum of (**5**) exhibited absorption bands at *ν* 1727 and 1684 cm^−1^, corresponding to two carbonyl groups. On the other hand, compound (**4**), as a typical 1,3-diketone, reacted with each hydrazine hydrate and thiourea to afford the corresponding 2-((3,5-dimethyl-1*H*-pyrazol-4-yl)thio)-7-phenyl-5-(quinolin-2-yl) pyrido[2,3-*d*]pyrimidin-4(3*H*)-one (**6**) and 2-((4,6-dimethyl-2-thioxo-1,2-dihydropyrimidin-5-yl)thio)-7-phenyl-5-(quinolin-2-yl) pyrido[2,3-*d*]pyrimidin-4(3*H*)-one (**7**), respectively. Infrared and nuclear magnetic resonance spectroscopy were used to confirm the chemical structures of compounds (**6**) and (**7**). The IR spectra of compound (**6**) showed a broad band at *ν* 3300, 3350, and 1690 cm^−1^, which corresponded to two groups of (2NH) and one carbonyl group (C=O), respectively. The ^1^H-NMR spectrum of compound (**6**) showed two singlet signals at *δ* 9.40 and 11.35 ppm, which came from two groups of two protons (2NH) and were exchangeable with D_2_O. Also, the ^1^H-NMR spectrum of compound (**7**) displayed two singlet signals at *δ* 11.50 and 11.77 ppm, originating from two groups of two protons (2NH) exchangeable with D_2_O. The ion peaks for compounds (**6** and **7**) in the mass spectrometry were identified at *m*/*z* = 476 (M^+^, 100%) and 520 (M^+^, 95%). The detailed spectral data for the new compounds are available in the Experimental Section, as shown in Scheme 2.

**Scheme 3:**
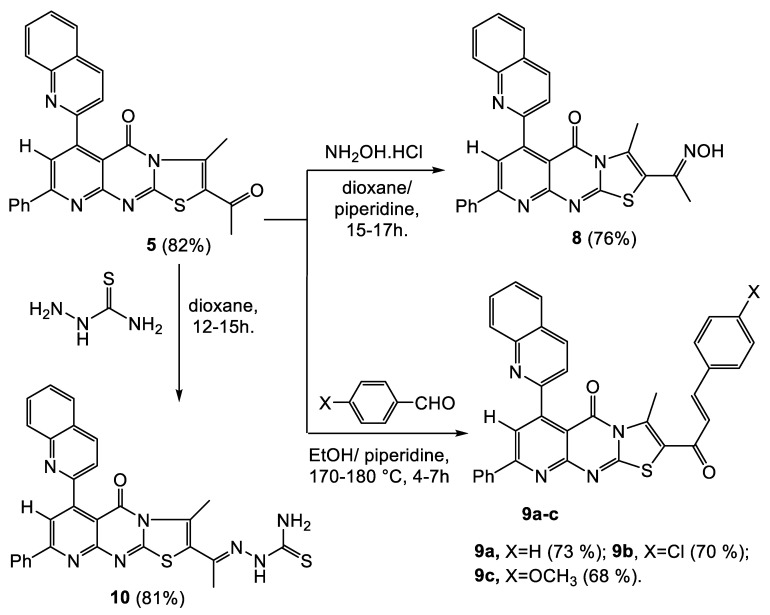
Synthesis of hydroxyimino, 4-sub-(phenyl) acryloyl, and hydrazine-1-carbothio-amide linked to quinoline-pyrido[2,3-*d*]thiazolo[3,2-*a*] pyrimidine derivatives.

Compound (**5**) showed characteristic reactions for the 2-acetyl group. When (**5**) reacted with hydroxylamine hydrochloride and thiosemicarbazide, it produced the corresponding oxime (**8**) and thiosemicarbazone (**10**) derivatives. Additionally, when compound (**5**) was heated with appropriate aromatic aldehydes at 180 °C in the presence of a small quantity of piperidine as a catalyst, it yielded 2-(3-(4-substituted-phenyl) acryloyl)-3-methyl-8-phenyl-6-(quinolin-2-yl)-5*H*-pyrido[2,3-*d*]thiazolo[3,2-*a*] pyrimidin-5-one (**9a**–**c**). The IR spectrum of (**8**) showed absorption bands at *ν* 3520 cm^−1^ for the hydroxyl group and 1692 cm^−1^ for the carbonyl group. Additionally, the ^1^H-NMR spectrum of (**8**) displayed a singlet signal at *δ* 11.95 ppm, indicating the presence of one proton of the (OH) groups (exchangeable with D_2_O). The compound (**9b**) IR spectrum showed broad bands for the two carbonyl groups at *ν* 1715 and 1691 cm^−1^. In the ^1^H-NMR spectrum of (**9b**), there were two doublet signals at *δ* 5.60 and 5.80 ppm, which corresponded to the four protons of the 4-chlorophenyl group, and a singlet signal at 8.25 ppm for the proton of the pyridine ring. The compound (**10**) IR spectrum showed two broadband absorptions at *ν* 3350–3410 cm^−1^ and 1681 cm^−1^ for the amino (NH) and amide carbonyl groups, respectively. The ^1^H-NMR spectrum of (**10**) revealed a singlet broad signal at *δ* 4.90 ppm corresponding to the (NH_2_) group and another singlet broad signal at *δ* 9.40 ppm confirming the (NH) group (exchangeable with D_2_O). In contrast, the presence of the amide carbonyl group was indicated by its ^13^C-NMR, showing a peak at *δ* 168.10 ppm. The MS of (**8**, **9a**–**c**, **10**) showed molecular ion peaks at *m*/*z* 477 (M^+^, 94%), 550 (M^+^, 97%), 585 (M^+^, 98%), 580 (M^+^, 96%), and 535 (M^+^, 100%), respectively. All spectroscopic analyses of the new chemical structures are detailed in Section 4 (Scheme 3).

**Scheme 4:**
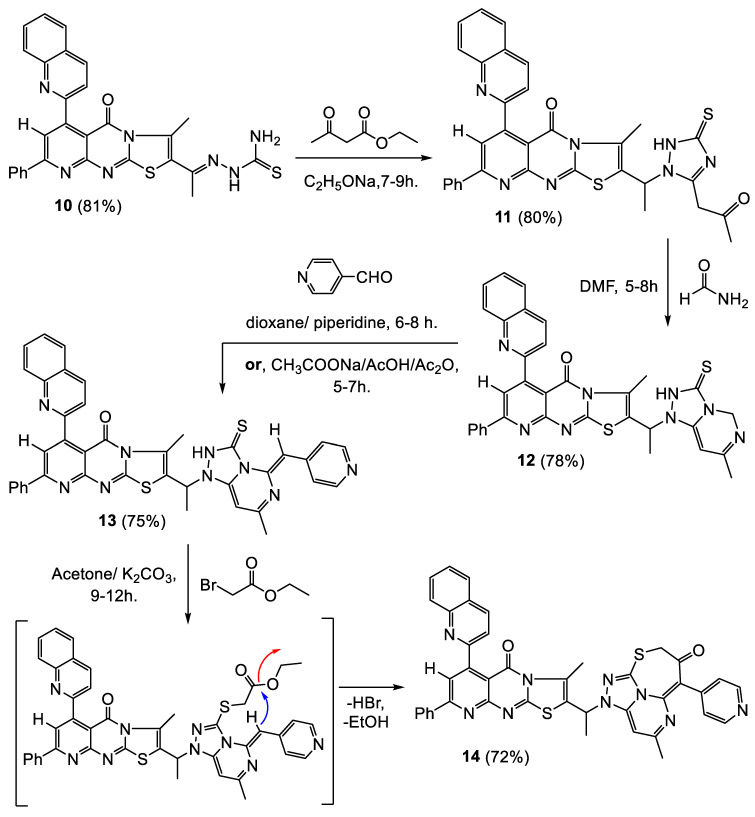
Synthesis of 1,3-thiazepinone and 1,2,4-triazolopyrimidine linked to quinoline-pyrido[2,3-*d*]thiazolo[3,2-*a*] pyrimidine derivatives (arrows illustrate the reaction mechanism and the formation direction of the product).

The treatment of compound (**10**) was conducted with ethyl acetoacetate in a sodium ethoxide solution under heat, stirring, and reflux for extended periods, resulting in the formation of 3-methyl-2-(1-(5-(2-oxopropyl)-3-thioxo-2,3-dihydro-1*H*-1,2,4-triazol-1-yl)ethyl)-8-phenyl-6-(quinolin-2-yl)-5*H*-pyrido[2,3-*d*]thiazolo[3,2-*a*]pyrimidin-5-one (**11**). The IR spectrum of compound (**11**) displayed absorption bands at wave numbers of *ν* 3250 cm^−1^ corresponding to one NH group and at 1693 and 1712 cm^−1^ corresponding to two carbonyl groups. Additionally, the ^1^H-NMR spectrum of compound (**11**) exhibited a singlet signal at a chemical shift of *δ* 8.76 ppm, corresponding to one NH group proton and being exchangeable with D_2_O. Furthermore, the reaction of (**11**) with formamide underwent long-term intramolecular cyclization in refluxing dimethylformamide to give 3-methyl-2-(1-(7-methyl-3-thioxo-2,3-dihydro-[1,2,4] triazolo[4,3-*c*] pyrimidin-1(5*H*)-yl)ethyl)-8-phenyl-6-(quinolin-2-yl)-5*H*-pyrido[2,3-*d*]thiazolo[3,2-*a*] pyrimidin-5-one (**12**). The IR spectrum of compound (**12**) showed absorption bands at *ν* 3310 cm^−1^ for the NH group and 1687 cm^−1^ for the carbonyl group. Additionally, the ^1^H-NMR spectrum of compound (**12**) revealed a singlet signal at *δ* 8.74 ppm for the proton of the NH group, which is exchangeable with D_2_O. Likewise, a mixture of compound (**12**) and isonicotinaldehyde in dioxane and piperidine as a catalyst was heated, refluxed, and stirred for a long time to form 3-methyl-2-(1-(7-methyl-5-(pyridin-4-ylmethylene)-3-thioxo-2,3-dihydro-[1,2,4]triazolo[4,3-*c*]pyrimidin-1(5*H*)-yl)ethyl)-8-phenyl-6-(quinolin-2-yl)-5*H*-pyrido[2,3-*d*]thiazolo[3,2-*a*] pyrimidin-5-one (**13**). Also, the latter compound (**13**) can be produced by reacting (**12**) with isonicotinaldehyde and anhydrous sodium acetate in glacial acetic acid/acetic anhydride. The mixture should be heated, stirred, and refluxed for extended times with TLC monitoring. In the IR spectrum of compound (**13**), there were broad bands at *ν* 3322 cm^−1^ for the (NH) group and one sharp intensity peak at ν 1693 cm^−1^ corresponding to the carbonyl group. The ^1^H-NMR spectrum of (**13**) showed three singlet signals at *δ* 8.08, 8.10, and 10.68 ppm for three protons of two protons of the pyridine and pyrimidine ring and one proton of the (NH) group, respectively. Alkylation was achieved by reacting compound (**13**) with ethyl-bromoacetate in dry acetone in the presence of potassium carbonate. The reaction was allowed to reflux for a long time. After removing hydrogen bromide and ethanol from the reaction mixture, 4-methyl-2-(1-(3-methyl-5-oxo-8-phenyl-6-(quinolin-2-yl)-5*H*-pyrido[2,3-*d*]thiazolo[3,2-*a*] pyrimidin-2-yl)ethyl)-6-(pyridin-4-yl)-2*H*-9-thia-1,2,2a1,5-tetraazabenzo[*cd*]azulen-7(8*H*)-one (**14**) was obtained. The chemical structure of (**14**) was confirmed by the ^1^H-NMR spectrum, which displayed several distinct signals at varying chemical shifts. Specifically, a doublet at *δ* 1.10 ppm represents three protons of a methyl group, a singlet at *δ* 2.24 ppm corresponds to another three protons of a different methyl group, and a singlet at *δ* 3.75 ppm indicates three protons of a third methyl group. A quartet at *δ* 4.01 ppm is attributed to one proton of a (CH) group. A singlet at *δ* 4.85 ppm also represents two protons of a (CH_2_) group within the thiazepine ring. Furthermore, a singlet at *δ* 8.03 ppm corresponds to one proton of a (CH) group in the pyrimidine, while a singlet at *δ* 8.10 ppm denotes one proton of a (CH) group in the pyridine. Its ^13^C-NMR of (**14**) shows five peaks at the following chemical shifts: at *δ* 37.00 ppm for one carbon atom of (CH_2_), 64.73 ppm for one carbon atom of (CH), 70.00 ppm for one carbon atom of the pyrimidine ring, 112.95 ppm for one carbon atom of the thiazole ring, 113.25 ppm for one carbon atom of the pyridine ring, and 164.4 and 168.0 ppm for the two carbon atoms of the carbonyl groups. The mass spectra of compounds (**11**, **12**, **13**, and **14**) showed molecular ion peaks at *m*/*z* = 603 (M^+^, 100%), 614 (M^+^, 98%), 703 (M^+^, 97%), and 743 (M^+^, 95%), respectively. The new chemical structures were confirmed through spectroscopic IR, ^1^H, ^13^C NMR, mass spectra, and elemental analysis, as shown in Section 4 and Scheme 4.

### 2.2. Biological Activities

#### 2.2.1. Biological Screening

All newly synthesized derivatives of quinoline-pyrido[2,3-*d*]thiazolo[3,2-*a*] pyrimidinone were tested for their antimicrobial activities in vitro at the lowest inhibitory concentration [32,76,77,78,79,80] against different types of bacteria (Gram-positive: *Streptococcus pyogenes*, *Staphylococcus aureus*; Gram-negative: *Klebsiella pneumoniae*, *Escherichia coli*) and fungi (*Candida albicans*, *Curvularia lunata*, *Alternaria alternate*, and A*spergillus niger*). Some of the compounds, such as **14**, **13**, **12**, **11**, and **10**, exhibited the best antibacterial and antifungal activity comparable to that of cefotaxime sodium (MIC = 1–2 μmol mL^−1^) and nystatin (MIC 1–3 μmol mL^−1^) as standard drugs, respectively. Some compounds, such as **9a**–**c**, **7**, **6**, **8**, and **5**, showed moderate antimicrobial activity, while the rest revealed weak antimicrobial activity. Detailed results are provided in Table 1 and Table 2.

#### 2.2.2. Structural Activity Relationship (SAR)

The results indicated that the prepared compounds displayed activity and sensitivity against various bacteria and fungi. Compounds (**10**–**14**) exhibited excellent antimicrobial activity as follows: 4-methyl-2-(1-(3-methyl-5-oxo-8-phenyl-6-(quinoline)-pyrido[2,3-*d*]thiazolo[3,2-*a*]pyrimidin-2-yl)ethyl)-6-(pyridin-4-yl)-9-thia-1,2,2a1,5-tetraazabenzo[*cd*] azulenone (**14**), 3-methyl-2-(1-(7-methyl-5-(pyridin-4-ylmethylene)-3-thioxo-2,3-dihydro-[1,2,4]triazolo[4,3-*c*]pyrimidin-1-yl)ethyl)-8-phenyl-6-(quinoline)-pyrido[2,3-*d*]thiazolo [3,2-*a*]pyrimidinone (**13**), 3-methyl-2-(1-(7-methyl-3-thioxo-[1,2,4]triazolo[4,3-*c*]pyramidin-1-yl)ethyl)-8-phenyl-6-(quinolin-2-yl)-pyrido[2,3-*d*]thiazolo[3,2-*a*]pyrimidinone (**12**), 3-methyl-2-(1-(5-(2-oxopropyl)-3-thioxo-2,3-dihydro-1,2,4-triazol-1-yl)ethyl)-8-phenyl-6-(quinolin-2-yl)-pyrido[2,3-*d*]thiazolo[3,2-*a*]pyrimidin-5-one (**11**), and 2-(1-(3-methyl-5-oxo-8-phenyl-6-(quinolin-2-yl)-pyrido[2,3-*d*]thiazolo[3,2-*a*]pyrimidin-2-yl)ethylidene)hydrazine-1-carbothioamide (**10**). Also, other compounds demonstrated moderate antimicrobial activity, such as (**9a**–**c**, **7**, **6**, **8** and **5**) as follows: 2-(3-(4-substituted-phenyl) acryloyl)-3-methyl-8-phenyl-6-(quinoline)-pyrido[2,3-*d*]thiazolo[3,2-*a*]pyrimidinone (**9a**–**c**), 2-((4,6-dimethyl-2-thioxo-pyrimidine)thio)-7-phenyl-5-(quinolin-2-yl)pyrido[2,3-*d*]pyrimidinone (**7**), 2-((3,5-dimethyl-pyrazol-4-yl)thio)-7-phenyl-5-(quinoline)pyrido[2,3-*d*] pyrimidinone (**6**), 2-(1-(hydroxyimino)ethyl)-3-methyl-8-phenyl-6-(quinoline)-pyrido[2,3-*d*]thiazolo[3,2-*a*]pyrimidinone (**8**), and 2-acetyl-3-methyl-8-phenyl-6-(quinolin-2-yl)-pyrido[2,3-*d*]thiazolo[3,2-*a*]pyrimidinone (**5**).

Therefore, after analyzing practical results and previous studies, it has been observed that certain compounds exhibit potent antimicrobial activity. This effectiveness is attributed to specific functional groups associated with quinoline-pyrido[2,3-*d*]thiazolo[3,2-*a*] pyrimidinone. These groups include 1,3-thiazepinone, 1,2,4-triazolopyrimidine, thiazole, pyridine, phenyl, chlorobenzenes, thioxo, amino, pyrazole, methoxybenzene, methyl, and acetyl moieties, along with heteroatoms such as oxygen, nitrogen, and sulfur. This class of heterocycles affects the types of bacteria and fungi encountered in this research. Previous scientific studies and references support these findings, showing similar results with these heterocyclic rings [32,50,51,52,53,54,55,56,57,58,59,60,61,62,63,64,65,66,67,68,69,70,76,77,78,79,80]. Additionally, past research and our current findings provide strong evidence for the promising antimicrobial activity of derivatives (**10**, **11**, **12**, **13**, and **14**). These compounds have significant potential for the further enhancement of their antimicrobial properties.

#### 2.2.3. Mechanism of Action

The time-kill test is often used to determine the bactericidal effect and is a valuable tool for understanding the dynamic interaction between the antimicrobial agent and the microbial strain. The time-kill examination shows whether the antimicrobial effect depends on time or concentration. This manuscript discusses the antibacterial properties and kinetic energy of quinoline-pyrido[2,3-*d*]thiazolo[3,2-*a*] pyrimidinone derivatives.

It was observed that these derivatives could significantly prolong the lag-phase growth of *Klebsiella pneumonia*, *E. coli*, *Streptococcus pyogenes*, and *Staphylococcus aureus* cells, extending the lag phase to approximately 10–14 h. Additionally, the derivatives were found to kill the cells within 2–4 h, as evidenced by transmission electron microscopy. The destruction of the cells’ outer and inner membranes is likely due to the penetration of the pyrido[2,3-*d*] pyrimidine derivatives, resulting in multiple holes and gaps in the damaged cells, ultimately leading to their destruction.

The following compounds have potential applications in the antimicrobial industry due to their antimicrobial properties: quinoline; pyrido[2,3-*d*]thiazolo[3,2-*a*]pyrimidine-pyridine-thiatetraazabenzoazulenone (**14**), 1,2,4-triazolo[4,3-*c*]pyrimidine-pyrido[2,3-*d*] thiazolo[3,2-*a*]pyrimidin-one (**13**), 1,2,4-triazolo[4,3-c]pyramidine-pyrido[2,3-*d*]thiazolo [3,2-a] pyrimidinone (**12**), 1,2,4-triazole-pyrido[2,3-*d*]thiazolo[3,2-*a*]pyrimidinone (**11**), and (**10**) derivatives.

In the time–kill assay, compounds (**10**–**14**) were found to destroy membrane integrity in all cells. Similarly, compounds **14**, **13**, and **12** were observed to act against food-borne and other pathogenic bacteria by destroying the cytoplasmic membrane.

Gram-negative bacteria have an outer membrane that is more complex and contains more lipopolysaccharide (LPS), which restricts the entry of hydrophobic compounds. On the other hand, Gram-positive bacteria lack the complex outer membrane and are surrounded by a thick peptidoglycan wall. This wall is not dense enough to block small antimicrobial molecules, making it easier for them to reach the cell membrane. Additionally, the lipophilic ends of lipoteichoic acid in the cell membrane of Gram-positive bacteria may aid in the penetration of hydrophobic compounds such as quinoline-pyrido[2,3-*d*]thiazolo[3,2-*a*] pyrimidinone derivatives.

The findings demonstrate that the antifungal activity of quinoline-pyrido[2,3-*d*]thiazolo[3,2-*a*] pyrimidinone derivatives against *Candida albicans*, *Curvularia lunata*, *Alternaria alternate*, and *Aspergillus niger* isolates is due to the inhibition of ergosterol biosynthesis and disruption of membrane integrity. Furthermore, a separate study highlights the potential for developing new formulations for candidiasis treatment, attributing the antifungal activity of these derivatives on *Candida albicans* to membrane damage, resulting in the leakage of intracellular components such as DNA. Additionally, perturbation of the fungal cell’s endomembrane system, including the mitochondria and plasma membrane, and the inhibition of ergosterol synthesis, mitochondrial ATPase, and dehydrogenase activities are associated with the antifungal effects of these derivatives on fungal cells [32,50,51,52,53,54,55,56,57,58,59,60,61,62,63,64,65,66,67,68,69,70,76,77,78,79,80].

## 3. Molecular Docking

Molecular docking is a widely utilized technique for predicting how small medicinal compounds interact with protein targets, including their affinity and effectiveness. This process is essential for rational drug design. Given the biological and pharmacological significance of docking studies, considerable efforts have been dedicated to improving algorithms that enhance the accuracy of these predictions [83]. Docking can also evaluate the interactions between synthesized compounds and protein receptors, providing insights into their binding mechanisms and potential antimicrobial properties [84,85,86].

### 3.1. Computational Methods

#### Molecular Docking of Synthesized Compounds

All protein receptors were obtained from the RCSB (Table 3). The structures of the target proteins were then preprocessed using PyMOL software (v2.5). The structure of the compound was drawn using BIOVIA Draw. Next, Open Babel [87] was employed to convert each compound into the mol2 format. AutoDock Tools were then used to transform the molecules into the pdbqt format. Before the docking process, ligand-centered maps were generated using AutoDock Vina [88]. The Discovery Studio program analyzed the 2D interactions between the targets and the ligands. Finally, the ADMET properties of the compounds were calculated using BIOVIA Discovery Studio software, version 17.2 (BIOVIA, 2017) [89].

### 3.2. Results and Discussions

#### 3.2.1. Docking and Interaction with Dihydropteroate Synthase of *S. aureus*

**Figure 3:**
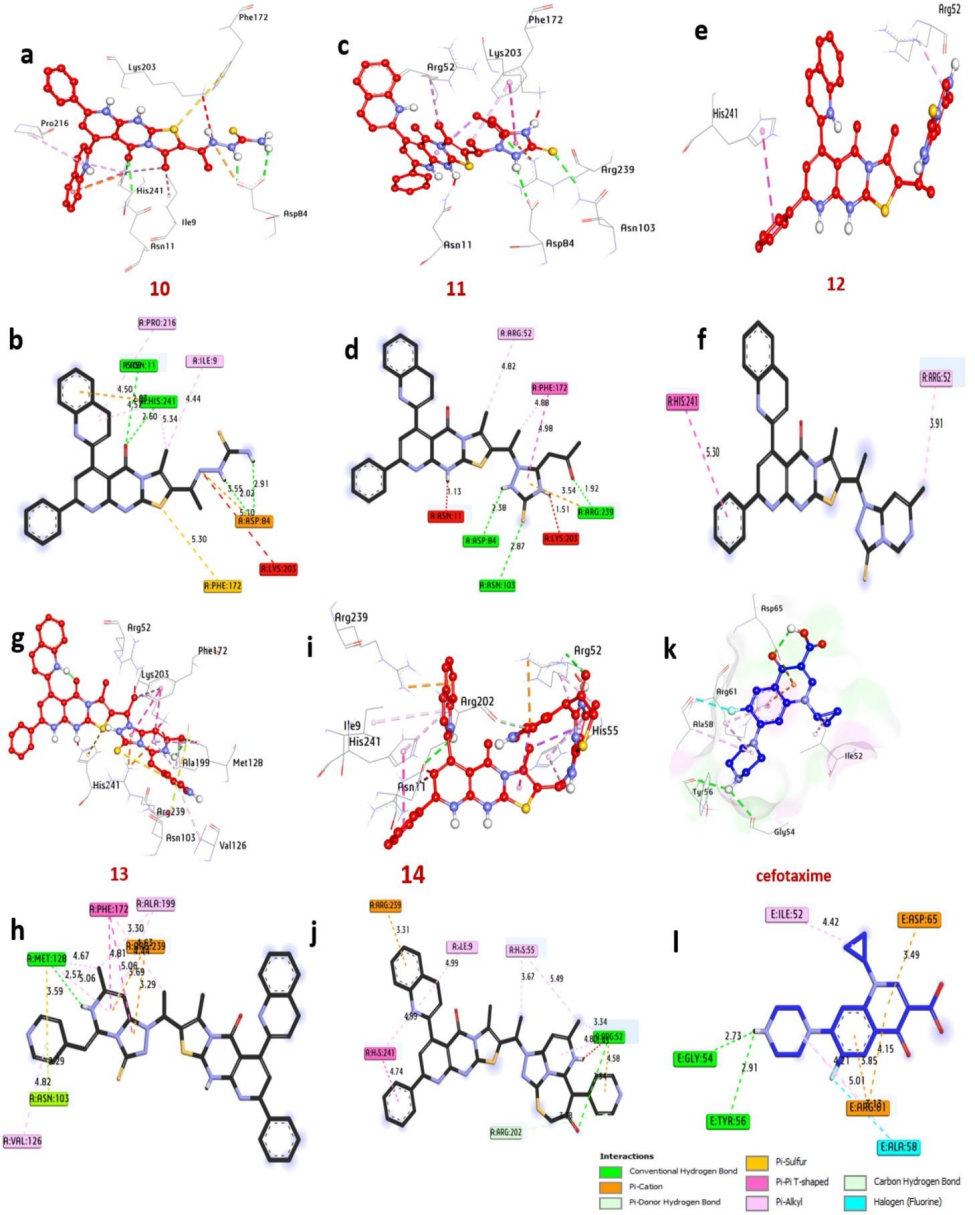
Three-dimensional representations of the compound at the binding pocket of dihydropteroate synthase of *S. aureus* (PDB: ID 1AD4): (**a**,**b**) **10**; (**c**,**d**) **11**; (**e**,**f**) **12**; (**g**,**h**) **13;** (**i**,**j**) **14;** and (**k**,**l**) cefotaxime.

Dihydropteroate synthase is an enzyme involved in the folate synthesis pathway, and its inhibition is essential for bacterial growth. Compounds **10**, **11**, **12**, **13**, and **14** demonstrated favorable binding energies of −8.90, −8.80, −8.70, −8.40, and −8.50 kcal/mol, respectively. In comparison, the binding energy of cefotaxime was −6.10 kcal/mol (see Table 4, Figure 3). These compounds formed hydrogen bonds with several amino acids, including Asn11, His241, Asp84, Arg239, Asn103, Met128, and Arg52. Additionally, various hydrophobic interactions were observed within the active pocket of the enzyme. These interactions included alkyl bonds with Ile9, Pro216, His241, Arg52, Phe172, Ala199, Met128, Val126, and His55, as well as pi–cation interactions with Arg239, Asp84, and His241. There were also pi–pi interactions with Phe172 and His241, pi–sulfur interactions with Met128 and Phe172, and CH-bonds with Arg202. Notably, the amino acids Asp84, Met128, and Arg52 located in the catalytic site increased the binding affinity of the compounds. Therefore, these compounds will likely exhibit antibacterial activity by inhibiting the dihydropteroate synthase enzyme in Staphylococcus aureus. This finding aligns with the research conducted in [90], which used molecular docking to assess the inhibitory interaction between a small molecule and the dihydropteroate synthase of *S. aureus*.

#### 3.2.2. Docking and Molecular Interaction Studies with DNA Gyrase of *E. coli*

**Figure 4:**
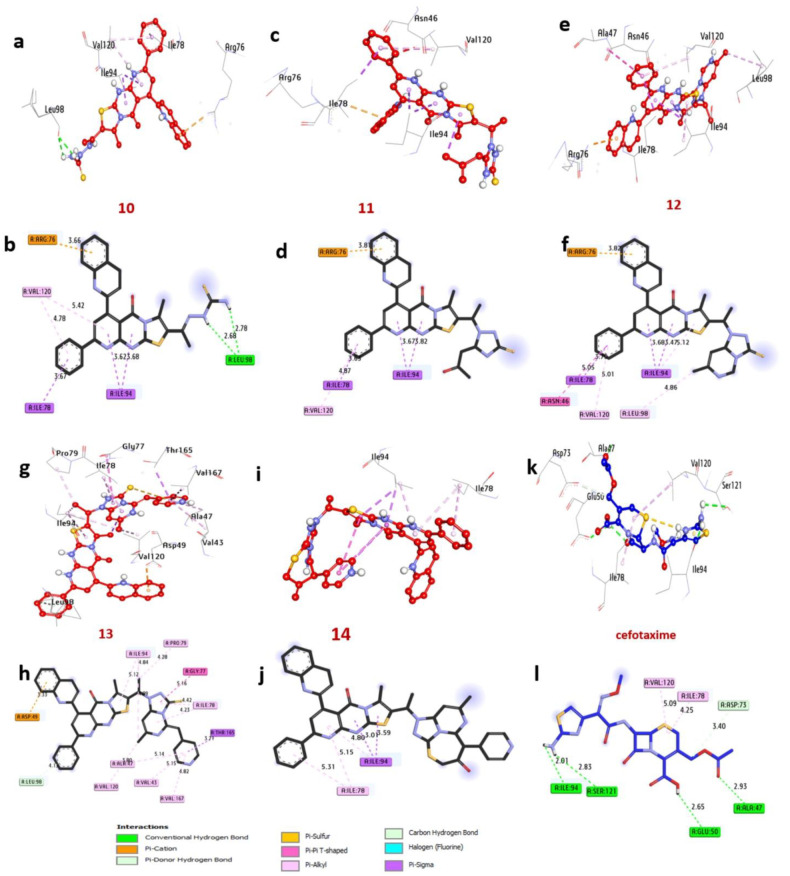
Three-dimensional representations of compounds at the binding pocket of DNA gyrase of *E. coli* (PDB: ID 7P2M): (**a**,**b**) **10**; (**c**,**d**) **11**; (**e**,**f**) **12**; (**g**,**h**) **13;** (**i**,**j**) **14;** and (**k**,**l**) cefotaxime.

DNA gyrase is an enzyme found in bacteria, including *E. coli*, that plays a crucial role in DNA replication and transcription. An analysis of docking results (see Table 5 and Figure 4) shows that compounds **10**, **11**, **12**, **13**, and **14** exhibited favorable binding energies of −8.90, −8.40, −8.10, −8.10, and −8.10 kcal/mol, respectively. In comparison, cefotaxime has a binding energy of −7.00 kcal/mol.

These compounds formed hydrogen bonds with vital amino acids, particularly Leu98. Several hydrophobic interactions occurred within the active site, including alkyl bonds with Ile94, Pro79, Ile78, Ala47, Val43, Val167, Val120, and Leu98. Other interactions included (pi–sigma) bonds with Ile94 and Ile78, (pi–cation) bonds with Arg76 and Asp49, (C-H) bonds with Leu98, and (pi–pi T-shaped) interactions with Gly77 and Asn46. Notably, the amino acids Leu98, Gly77, and Ile78 in the catalytic site enhance the binding affinity.

The authors of [86] reported similar findings. They investigated the antimicrobial activity of the identified compounds against *E. coli* and validated the inhibition by performing in silico interactions between the isolated ligands and DNA gyrase, a crucial protein receptor.

#### 3.2.3. Docking and Molecular Interaction Studies of Sortase A in *S. pyogenes*

**Figure 5:**
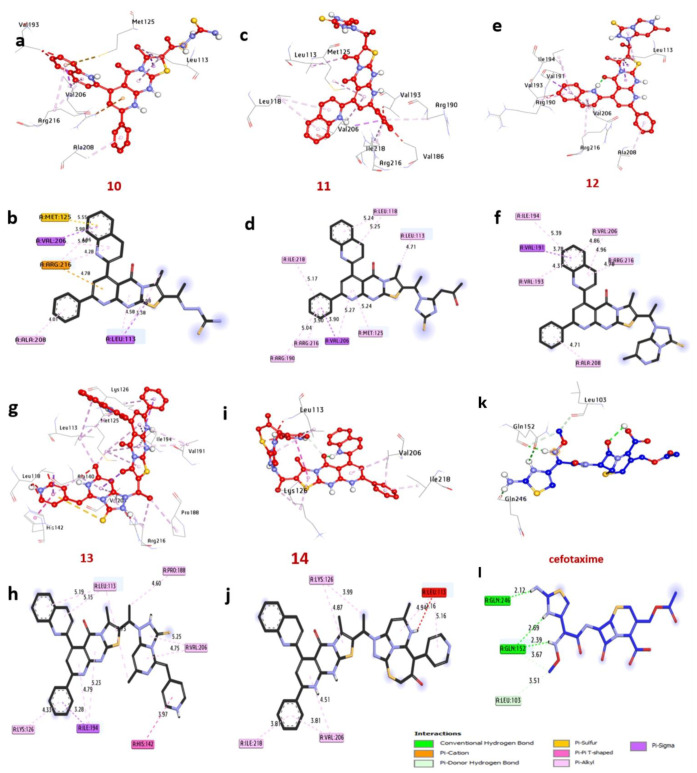
Three-dimensional representations of the compound at the binding pocket of Sortase A in *S. pyogenes* (PDB: ID 8T8G): (**a**,**b**) **10**; (**c**,**d**) **11**; (**e**,**f**) **12**; (**g**,**h**) **13**; (**i**,**j**) **14**; and (**k**,**l**) cefotaxime.

Sortase A is an enzyme that plays a crucial role in anchoring surface proteins to the cell wall, a process essential for the virulence of *Streptococcus pyogenes*. Among the tested compounds, compounds **10**, **11**, **12**, **13**, and **14** demonstrated favorable binding energies of −8.20, −7.90, −7.80, −7.90, and −8.00 kcal/mol, respectively. In comparison, the binding energy of cefotaxime was −7.30 kcal/mol. Notably, these compounds did not form hydrogen bonds. Several hydrophobic interactions were identified within the active site, including alkyl bonds with residues such as Arg216, Ala208, Ile194, Val193, Val206, Pro188, Leu113, Lys126, Leu11, and Ile218. Additionally, pi–sulfur interactions involved Met125, while pi–cation interactions occurred with Arg216. There were also pi–pi T-shaped interactions with His142 and pi–sigma interactions with Val206, Leu113, Val191, and Ile194. Furthermore, residues Arg216, Gln152, and Gln246 at the catalytic site were found to enhance the binding affinity of these compounds. Coupled with their demonstrated in vitro antibacterial activity, these findings suggest that these compounds could serve as potent bacterial inhibitors. These results align with work [84], which used molecular docking to investigate the inhibitory interaction between a small molecule and the LasR protein in *S. pyogenes* (see Table 6 and Figure 5).

#### 3.2.4. Docking and Interaction of KPC-2 Carbapenemase of *K. pneumoniae*

**Figure 6:**
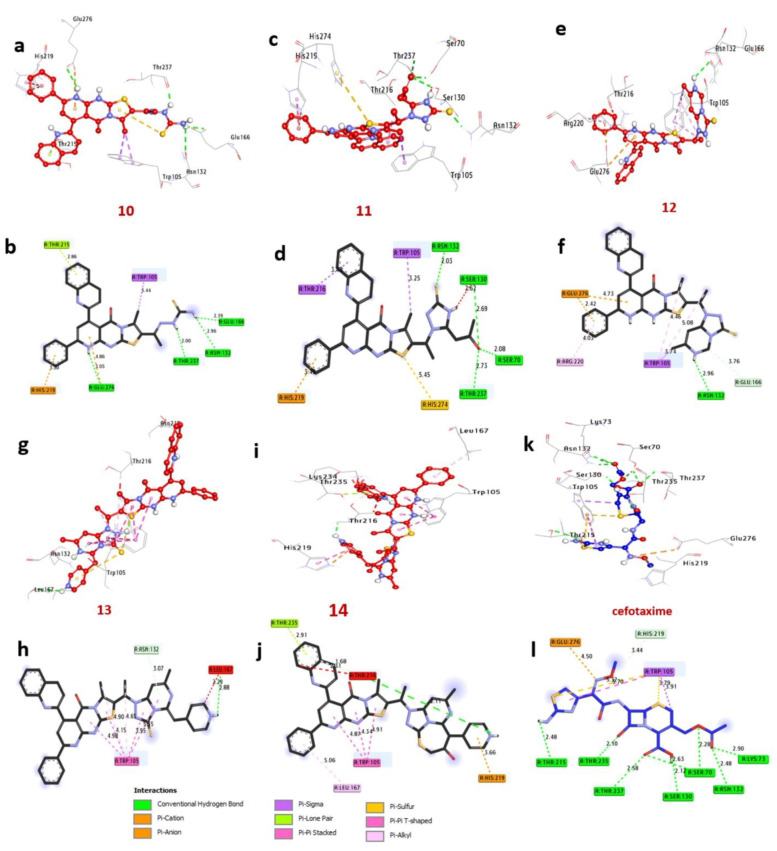
Three-dimensional representations of compounds at the binding pocket of KPC-2 carbapenemase of *K. pneumoniae* (PDB: ID 2OV5): (**a**,**b**) **10**; (**c**,**d**) **11**; (**e**,**f**) **12**; (**g**,**h**) **13**; (**i**,**j**) **14**; and (**k**,**l**) cefotaxime.

KPC-2 carbapenemase is a crucial enzyme found in *Klebsiella pneumoniae*, which provides resistance to carbapenem antibiotics, a class used to treat multidrug-resistant bacterial infections. The docking findings with the selected molecules are summarized in Table 7 and Figure 6. Among all the compounds tested, compounds 10, 11, 12, 13, and 14 demonstrated favorable binding energies of −8.00, −8.40, −7.90, −7.80, and −8.10 kcal/mol, respectively, in comparison to cefotaxime, which had a binding energy of −8.40 kcal/mol. The compounds formed hydrogen bonds with key amino acids, including Glu166, Asn132, Thr237, Glu276, Ser130, Leu167, and Thr216. Several hydrophobic interactions occurred within the active site, such as alkyl bonds with Trp105, Arg220, and Leu167. There were also (pi–cation) interactions with Glu276, (C-H bond) interactions with Glu166 and Asn132, and (pi–pi T-shaped) interactions with Trp105. Other interactions included the pi–lone pair ones with Thr215 and Thr235; (pi–cation) interactions with His219 and Glu276; and (pi–sigma) interactions with His274, Thr216, and Trp105. Notably, the amino acids Glu166, Leu167, and Ser70 in the catalytic site significantly enhance binding affinity. Furthermore, the findings, corroborated by in vitro antibacterial activity results, suggest that these compounds may serve as potent bacterial inhibitors [91], and similar results were reported when testing the antibacterial activity of these identified compounds against *K. pneumoniae*.

#### 3.2.5. Docking and Interaction with Sterol 14-Alpha Demethylase of *C. albicans*

**Figure 7:**
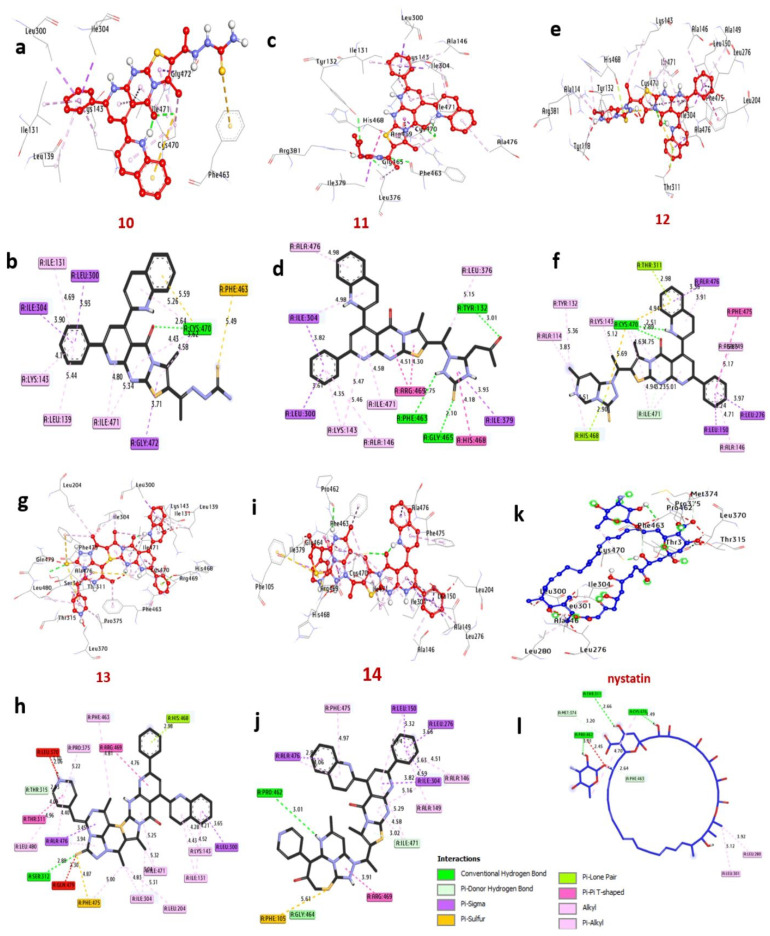
Three-dimensional representations of compound conformations at the binding pocket of sterol 14-alpha demethylase of *C. albicans* (PDB: ID 5TZ1): (**a**,**b**) **10**; (**c**,**d**) **11**; (**e**,**f**) **12**; (**g**,**h**) **13**; (**i**,**j**) **14**; and (**k**,**l**) nystatin.

Sterol 14-alpha demethylase is an enzyme that plays a crucial role in synthesizing ergosterol, an essential component of the cell membrane in *Candida albicans*. Inhibiting this enzyme results in cell membrane dysfunction, ultimately leading to fungal cell death. The docking study results with the selected molecules are presented in Table 8 and Figure 7. Among all the compounds tested, compounds **10**, **11**, **12**, **13**, and **14** exhibited the highest affinity interactions, with binding energies of −10.20, −9.90, −9.00, and −8.80 kcal/mol, respectively, compared to nystatin, which has a binding energy of −7.20 kcal/mol. These compounds form hydrogen bonds with key amino acids, including Cys470, Tyr132, Phe463, Gly465, Ser312, and Pro462. Additionally, several hydrophobic interactions occur within the active site pocket. These include alkyl bonds with amino acids like Ile131, Leu139, Lys143, Ile471, Cys470, Leu376, Ala476, Ile304, Tyr132, Ala114, Ala146, Phe463, Pro375, Leu480, Leu204, and Phe475. Moreover, various types of interactions were observed, such as (pi–sigma) interactions with Ala476, Leu150, Ile379, Ile304, Leu300, Gly472, and Leu276; (pi–pi T-shaped) interactions with Arg469, His468, and Thr311; (carbon–H) bonds with Thr315 and Ile471; and (pi–sulfur) bonds with Phe105 and Phe463. Furthermore, it is important to note that the amino acids Cys470, Tyr132, and Phe463, located in the catalytic site, enhance the binding affinity of these compounds.

#### 3.2.6. Docking and Molecular Interaction with fdc1of *Aspergillus niger* (PDB: ID 4ZA5)

**Figure 8:**
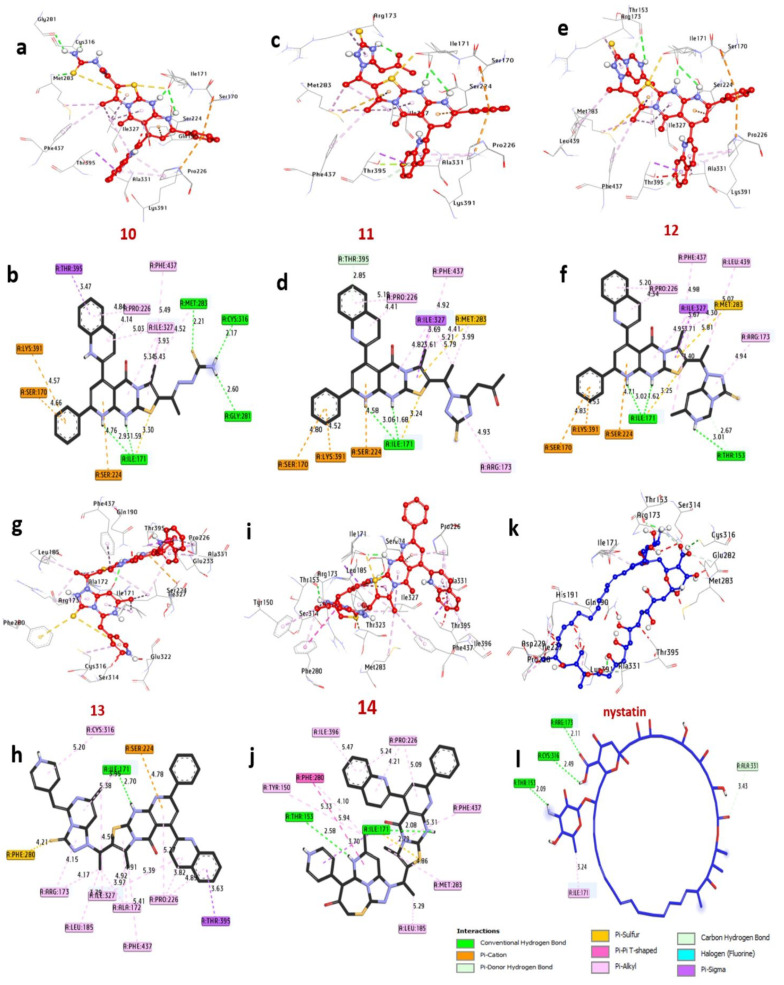
Three-dimensional representations of compound conformations at the binding pocket of the fdc1of *A. niger* (PDB: ID 4ZA5): (**a**,**b**) **10**; (**c**,**d**) **11**; (**e**,**f**) **12**; (**g**,**h**) **13;** (**i**,**j**) **14;** and (**k**,**l**) nystatin.

The FDC1 protein is associated with the breakdown of aromatic compounds in *Aspergillus niger*, as shown in Table 9 and Figure 8, playing a crucial role in producing the distinctive volatile aromas found in fermented beverages like beer and wine. Docking analysis has shown that compounds **10**, **11**, **12**, **13**, and **14** have binding energies of −11.70, −11.60, −10.10, −10.50, and −117 kcal/mol, respectively, which are significantly better than nystatin’s binding energy of −8.58 kcal/mol. These compounds form essential hydrogen bonds with key amino acids, including Ile171, Met283, Cys316, Gly281, and Thr153, which enhances their interactions. Additionally, non-hydrophilic interactions—such as alkyl bonds with various amino acids, pi–sigma bonding, pi–sulfur interactions, amid–pi stacking, carbon–H bonding, and pi–cation interactions—contribute to a complex binding network. Significantly, the residues Ile171, Gln190, and Ser223 located within the catalytic site positively affect the binding affinity of these compounds. In conclusion, the data strongly indicate that compounds **10**, **11**, **12**, **13**, and **14** have significant potential for further investigation as inhibitors of fungal FDC1 in *A. niger*.

#### 3.2.7. Docking and Interaction with AaTPS of *Alternaria alternata*

**Figure 9:**
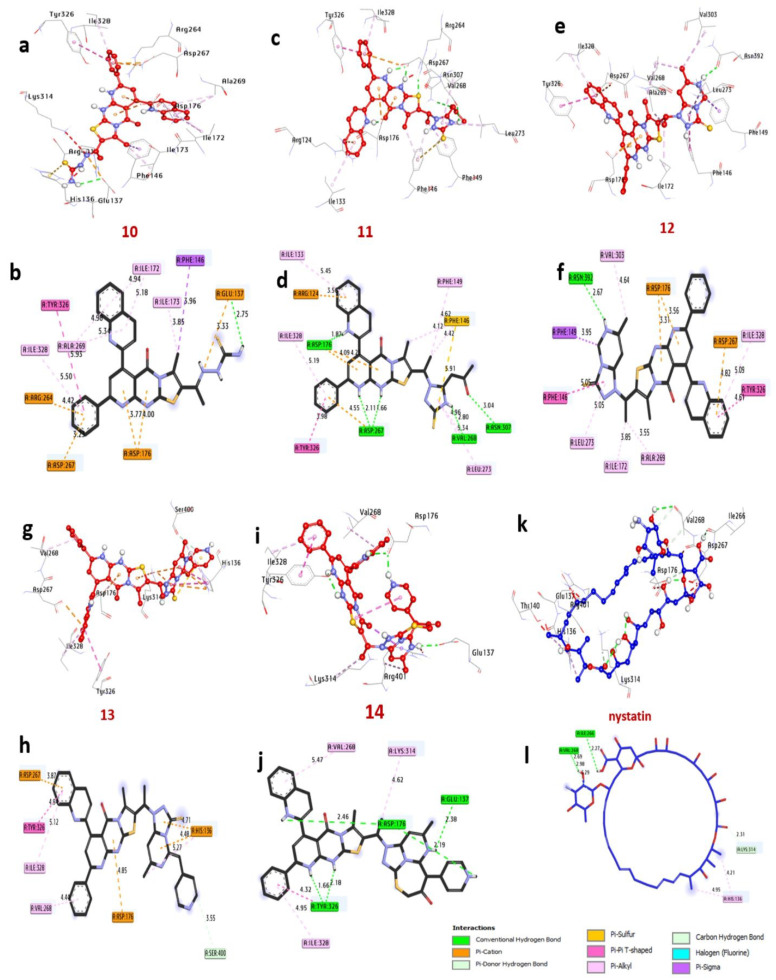
Three-dimensional representations of compound conformations at the binding pocket of AaTPS of *Alternaria alternata* (PDB: ID 6LCD): (**a**,**b**) **10**; (**c**,**d**) **11**; (**e**,**f**) **12**; (**g**,**h**) **13**; (**i**,**j**) **14** and (**k**,**l**) nystatin.

The docking findings with the selected molecules are presented in Table 10 and Figure 9. Among all the compounds, compounds **10**, **11**, **12**, **13**, and **14** exhibited the most robust affinity interactions, with binding energies of −10.20, −9.90, −9.00, −8.80, and −7.20 kcal/mol, respectively, compared to nystatin, which has a binding energy of −7.00 kcal/mol. These compounds formed hydrogen bonds with vital amino acids, including Glu137, Asp176, Asp267, Val268, Asn307, Asn392, and Tyr326. Additionally, several hydrophobic interactions occurred within the active site, including alkyl bonds with Ile173, Ile172, Ala269, Ile328, Phe149, Ile133, Leu273, Val303, His136, and Lys314. Other interactions included pi–cation bonds with Asp176, Asp267, Arg264, Glu137, His136, and Arg124; amid–pi stacking with Tyr326; pi–sigma interactions with Phe146 and Phe149; pi–sulfur interactions with Phe146; and carbon-hydrogen bonds with Lys314. Furthermore, the amino acids Glu137, Asp176, and Asp267 in the catalytic site significantly enhanced the binding affinity.

## 4. Experimental Section

Cooperating with other researchers, a research plan was created to synthesize new heterocyclic compounds from quinoline and pyridopyrimidine derivatives. These compounds were intended for studying their antimicrobial activity, and the plan was successfully executed.

### 4.1. General Information

All melting points were determined using an Electrothermal IA 9100 series digital melting point apparatus (Shimadzu, Tokyo, Japan). Elemental analyses were conducted on Vario EL (Elementar, Langenselbold, Germany). The microanalytical data were processed at the microanalytical center of the Faculty of Science at Cairo University and the National Research Centre. The IR spectra (KBr disc) were recorded using a Perkin-Elmer 1650 spectrometer (Waltham, MA, USA). NMR spectra were obtained using JEOL 270 MHz and JEOL JMS-AX 500 MHz (JEOL, Tokyo, Japan) spectrometers with (Me_4_Si) as an internal standard. Mass spectra were recorded on an EI Ms-QP 1000 EX instrument (Shimadzu, Tokyo, Japan) at 70 eV. Biological evaluations were carried out by the antimicrobial unit of the Department of Chemistry of Natural and Microbial Products (National Research Centre, Giza, Egypt). All starting materials and solvents were purchased from Sigma-Aldrich (St. Louis, MO, USA).

### 4.2. Synthesis of 7-Phenyl-5-(quinolin-2-yl)-2-thioxo-2,3-dihydropyrido[2,3-d] Pyrimidin-4(1H)-one (**1**) [32,40]

General Procedure [32,40]: A mixture of 1-phenyl-3-(quinoline) propenone (chalcone, 2.59 g, 0.01 mol) and 6-amino-2-thioxopyrimidinone (1.43 g, 0.01 mol) in 30 mL of dimethylformamide was heated under reflux for 18–20 h. Once the reaction was complete and confirmed by TLC, the solution was cooled to 0–5 °C, and the resulting solid was filtered out and recrystallized from DMF. The final product (**1**) was obtained as yellow crystals with an 85% yield.

### 4.3. Synthesis of 2-((2-Oxo-substituted)thio)-7-phenyl-5-(quinolin-2-yl) Pyrido[2,3-d] Pyrimidin-4(3H)-one (**2a**, **b**)

General Procedure: To a warmed ethanolic sodium hydroxide solution [prepared by dissolving (0.4 g, 10 mmol) of NaOH in 50 mL ethanol], compound 1 (3.82 g, 10 mmol) was added, the heating was continued for 40 min, and the mixture was allowed to cool to room temperature. The proper α--halo ketones, such as chloroacetone and phenacyl bromide (0.012 mol), were added. The mixture was stirred under reflux for 5–7 h, cooled to room temperature, and poured into cold water (100 mL). The solid product precipitated and was filtered off, washed with 100 mL water, dried, and crystallized to produce (**2a**, **b**).

### 4.4. Synthesis of 2-((2-Oxopropyl)thio)-7-phenyl-5-(quinolin-2-yl) Pyrido[2,3-d] Pyrimidin-4(3H)-one (**2a**)

The yellow crystals were obtained from (1) and chloroacetone (0.92 mL, 10 mmol). The compound was crystallized from methanol with a 90% yield and had a melting point of >350 °C (dec.); IR (*ν*, cm^−1^) KBr: 3250 (NH), 3095 (CH aryl), 2975 (CH alkyl), 1720 (CO, ketone), 1690 (CO, amide), 1640 (C=N), 1590 (C=C); ^1^H NMR (DMSO-*d6*, ppm) *δ* 2.20 (s, 3H, CH_3_), 4.10 (s, 2H, CH_2_), 7.10–7.75 (m, 11H, phenyl, quinoline), 8.20 (s, 1H, pyridine), 9.10 (br., 1H, NH, D_2_O exchangeable); ^13^C NMR (DMSO-*d*_6_) *δ* 24.5 (1C, CH_3_), 36.7 (1C, CH_2_), 115.1 (1C, CH, pyridine), 117.8, 119.1, 120.4, 124.9, 127.1, 127.2, 127.7, 128.2, 129.1, 130.1, 138.8, 139.1, 143.7, 150.5, 153.9, 154.6, 155.8, 158.9 (20C, Ar-C), 162.1, 190.5 (2C, two carbonyl group); and MS (70 ev, %): *m*/*z* = 438 (M^+^, 100%). Anal. Calc. for C_25_H_18_N_4_O_2_S (438.51): C, 68.48 (68.55); H, 4.14 (4.10); and N, 12.78 (12.71).

### 4.5. Synthesis of 2-((2-Oxo-2-phenylethyl)thio)-7-phenyl-5-(quinolin-2-yl) Pyrido[2,3-d] Pyrimidin-4(3H)-one (**2b**)

The yellowish crystals were obtained from (1) and phenacyl bromide (1.99 g, 10 mmol). The compound was crystallized from ethanol with a 88% yield and had a melting point of >350 °C (dec.); IR (*ν*, cm^−1^) KBr: 3255 (NH), 3090 (CH aryl), 2972 (CH alkyl), 1725 (CO, ketone), 1695 (CO, amide), 1635 (C=N), 1594 (C=C); ^1^H NMR (DMSO-*d6*, ppm) *δ* 4.16 (s, 2H, CH_2_), 6.97–7.92 (m, 16H, two phenyl, quinoline), 8.08 (s, 1H, pyridine), 8.75 (br., 1H, NH, D_2_O exchangeable); ^13^C NMR (CDCl_3,_ ppm) *δ* 37.0 (1C, CH_2_), 113.5 (1C, CH, pyridine), 113.7, 114.1, 114.3, 116.8, 121.9, 122.0, 122.1, 122.2, 125.8, 126.0, 128.9, 129.1, 129.6, 129.7, 130.1, 130.3, 137.7, 145.4, 145.6, 146.4, 146.9, 147.1, 155.8, 161.7, 161.9, 164.2 (26C, Ar-C), 164.4, 167.9 (2C, two carbonyl group); and MS (70 ev, %): *m*/*z* = 500 (M^+^, 100%). Anal. Calc. for C_30_H_20_N_4_O_2_S (500.58): C, 71.98 (71.92); H, 4.03 (4.10); and N, 11.19 (11.26).

### 4.6. Synthesis of 3-Substituted-8-phenyl-6-(quinolin-2-yl)-5H-pyrido[2,3-d]thiazolo[3,2-a] Pyrimidin-5-one (**3a**, **b**)

A solution of **2a**, **b** (10 mmol) in glacial acetic acid (45 mL) and a catalytic amount of sulfuric acid (1 mL) was stirred under reflux for 8–10 h. The reaction mixture was cooled, poured into cold water (100 mL), and neutralized by an ammonia solution. The solid precipitate was filtered off, washed with water, dried, and crystallized to produce (**3a**, **b**).

### 4.7. Synthesis of 3-Methyl-8-phenyl-6-(quinolin-2-yl)-5H-pyrido[2,3-d]thiazolo[3,2-a] Pyrimidin-5-one (**3a**)

The yellowish crystals were obtained from **2a** (4.38 g, 10 mmol). The compound was crystallized from dimethylformamide with an 86% yield and had a melting point of >350 °C (dec.); IR (*ν*, cm^−1^) KBr: 3075 (CH aryl), 2960 (CH alkyl), 1685 (CO, amide), 1642 (C=N), 1580 (C=C); ^1^H NMR (DMSO-*d6*, ppm) *δ* 2.23 (s, 3H, CH_3_), 4.90 (s, 1H, thiazole), 7.10–7.99 (m, 11H, phenyl, quinoline), 8.01 (s, 1H, pyridine); ^13^C NMR (DMSO-*d*_6_) *δ* 21.2 (1C, CH_3_), 97.5 (1C, CH, thiazole), 118.5 (1C, CH, pyridine), 119.1, 120.4, 121.7, 125.3, 127.2, 127.4, 127.8, 128.1, 129.3, 129.8, 138.2, 139.1, 141.1, 144.5, 150.4, 154.1, 155.3, 156.1, 158.5 (21C, Ar-C), 166.2 (1C, one carbonyl group); and MS (70 ev, %): *m*/*z* = 420 (M^+^, 100%). Anal. Calc. for C_25_H_16_N_4_OS (420.49): C, 71.41 (71.37); H, 3.84 (3.88); and N, 13.32 (13.28).

### 4.8. Synthesis of 3,8-Diphenyl-6-(quinolin-2-yl)-5H-pyrido[2,3-d]thiazolo[3,2-a] Pyrimidin-5-one (**3b**)

The pale-yellow crystals were obtained from **2b** (5.00 g, 10 mmol). The compound was crystallized from dioxane with an 82% yield and had a melting point of >350 °C (dec.); IR (*ν*, cm^−1^) KBr: 3072 (CH aryl), 2964 (CH alkyl), 1682 (CO, amide), 1644 (C=N), 1582 (C=C); ^1^H NMR (DMSO-*d6*, ppm) *δ* 6.15 (s, 1H, thiazole), 7.01–7.97 (m, 16H, two phenyl, quinoline), 8.25 (s, 1H, pyridine); ^13^C NMR (DMSO-*d*_6_) *δ* 107.1 (1C, CH, thiazole), 119.3 (1C, CH, pyridine), 119.6, 120.9, 121.2, 124.8, 127.1, 127.2, 127.5, 127.8, 128.2, 128.4, 128.7, 129.1, 129.7, 134.1, 137.5, 139.3, 144.5, 148.7, 150.6, 154.2, 155.4, 156.3, 158.7 (27C, Ar-C), 167.1 (1C, one carbonyl group); and MS (70 ev, %): *m*/*z* = 482 (M^+^, 100%). Anal. Calc. for C_30_H_18_N_4_OS (482.56): C, 74.67 (74.62); H, 3.76 (3.80); and N, 11.61 (11.66).

### 4.9. Synthesis of 3-((4-Oxo-7-phenyl-5-(quinolin-2-yl)-3,4-dihydropyrido[2,3-d] Pyrimidin-2-yl)thio) Pentane-2,4-dione (**4**)

A warmed ethanolic sodium hydroxide solution (prepared by dissolving 10 mmol of NaOH in 50 mL ethanol) was added to each one (3.82 g, 10 mmol), the heating was continued for 35 min, and the mixture was allowed to cool to room temperature. The proper 3-chloro-pentane-2,4-dione (0.012 mol) was added. The mixture was stirred under reflux for 5–7 h, cooled to room temperature, and poured into cold water (100 mL). The solid product precipitated was filtered off and washed with 100 mL water; the product was dried and crystallized from dimethylformamide as yellowish crystals, in 84% yield, with a melting point of >350 °C (melted); IR (*ν*, cm^−1^) KBr: 3270 (NH), 3085 (CH aryl), 2970 (CH alkyl), 1724, 1719 (2CO, ketone), 1681 (CO, amide), 1643 (C=N), 1585 (C=C); ^1^H NMR (DMSO-*d6*, ppm) *δ* 2.80, 3.31 (2s, 6H, 2CH_3_), 5.13 (s, 1H, CH), 6.92–7.85 (m, 11H, phenyl, quinoline), 8.01 (s, 1H, pyridine), 10.68 (br., 1H, NH, D_2_O exchangeable); ^13^C NMR (CDCl_3,_ ppm) *δ* 21.17, 21.27 (2C, 2CH_3_), 59.80 (1C, CH), 100.13 (1C, CH, pyridine), 104.5, 113.1, 116.2, 125.7, 125.9, 126.5, 126.8, 128.9, 129.0, 129.1, 129.4, 135.9, 136.8, 138.2, 139.9, 141.3, 147.3, 156.2 (20C, Ar-C, 20 sp^2^ carbon with four symmetric carbon atoms), 168.5 (1C, one amide carbonyl group); 194.4 (2C, two ketone carbonyl group); and MS (70 ev, %): *m*/*z* = 480 (M^+^, 100%). Anal. Calc. for C_27_H_20_N_4_O_3_S (480.54): C, 67.49 (67.55); H, 4.20 (4.25); N, 11.66 (11.71).

### 4.10. Synthesis of 2-Acetyl-3-methyl-8-phenyl-6-(quinolin-2-yl)-5H-pyrido[2,3-d]thiazolo[3,2-a] Pyrimidin-5-one (**5**)

Solution of compound **4** (4.80 g, 10 mmol) in an acetic anhydride-pyridine (20:10 mL) mixture was stirred under reflux for 6 h. The reaction mixture was allowed to cool to room temperature. Then, it was poured into cold water (100 mL), and the deposited precipitate was filtered off, dried, and crystallized from methanol. The compound was obtained as yellow crystals in 82% yield, m. p. > 350 °C (melted); IR (*ν*, cm^−1^) KBr: 3072 (CH aryl), 2960 (CH alkyl), 1727 (CO, ketone), 1684, (CO, amide), 1638 (C=N); 1579 (C=C); ^1^H NMR (DMSO-*d6*, ppm) *δ* 2.79 (s, 3H, CH_3_), 2.83 (s, 3H, CH_3_), 6.91–7.99 (m, 11H, phenyl, quinoline), 8.13 (s, 1H, pyridine); ^13^C NMR (DMSO-*d*_6_) *δ* 16.50, 24.20 (2C, 2CH_3_), 115.7 (1C, CH, pyridine), 116.1, 117.8, 119.5, 120.7, 124.6, 126.9, 127.2, 127.8, 128.4, 129.1, 129.7, 135.5, 138.4, 144.1, 145.9, 150.5, 154.3, 154.8, 156.1, 157.9 (22C, Ar-C, 22 sp^2^ carbon with four symmetric carbon atoms), 164.20, 186.40 (2C, two carbonyl groups); and MS (70 ev, %): *m*/*z* = 462 (M^+^, 100%). Anal. Calc. for C_27_H_18_N_4_O_2_S (462.53): C, 70.11 (70.16); H, 3.92 (3.85); N, 12.11 (12.17).

### 4.11. Synthesis of 2-((3,5-Dimethyl-1H-pyrazol-4-yl)thio)-7-phenyl-5-(quinolin-2-yl)pyrido[2,3-d] Pyrimidin-4(3H)-one (**6**)

A mixture of **4** (4.80 g, mmol) and hydrazine hydrate (99–100%) in dioxane (30 mL) and ethanol (20 mL) was stirred under reflux for 15 h. The reaction mixture was allowed to cool to room temperature and poured into cold water (100 mL). The deposited precipitate was filtered off, dried as white crystals, and crystallized from methanol/dioxane (1:1), which yielded a 78% yield. M. p. > 350 °C (melted); IR (*ν*, cm^−1^) KBr: 3300, 3350 (2NH), 3070 (CH aryl), 2960 (CH alkyl), 1690 (CO, amide), 1638 (C=N), 1577 (C=C); ^1^H NMR (DMSO-*d6*, ppm) *δ* 1.4, 1.5 (2s, 6H, 2CH_3_), 6.60–7.72 (m, 11H, phenyl, quinoline), 8.07 (s, 1H, pyridine), 9.40, 11.35 (2brs, 2NH, D_2_O exchangeable); ^13^C NMR (DMSO-*d*_6_) *δ* 15.9 (2C, 2CH_3_), 114.8 (1C, CH, pyridine), 115.1, 117.4, 118.5, 120.1, 123.6, 126.2, 127.1, 127.5, 128.4, 129.3, 129.7, 135.5, 138.1, 142.6, 143.8, 149.7, 153.5, 153.9, 155.3, 158.5 (23C, Ar-C, 23 sp^2^ carbon with four symmetric carbon atoms), 162.10 (1C, one carbonyl group); MS (70 ev, %): *m*/*z* = 476 (M^+^, 100%); Anal. Calc. for C_27_H_20_N_6_OS (476.14): C, 68.05 (68.11); H, 4.23 (4.28); N, 17.64 (17.55).

### 4.12. Synthesis of 2-((4,6-Dimethyl-2-thioxo-1,2-dihydropyrimidin-5-yl)thio)-7-phenyl-5-(quinolin-2-yl) Pyrido[2,3-d] Pyrimidin-4(3H)-one (**7**)

A mixture of 4 (4.80 g, mmol) and thiourea (0.76 g, 10 mmol) was stirred under reflux in dioxane (30 mL) in the presence of the catalytic amount of piperidine for 18 h. The reaction mixture was allowed to cool to room temperature and poured into water (100 mL). The deposited precipitate was filtered off, washed with ethanol (40 mL), dried, and crystallized from dimethylformamide as a yellowish powder in 75% yield, m. p. > 350 °C (melted); IR (*ν*, cm^−1^) KBr: 3320, 3360 (2NH), 3065 (CH aryl), 2955 (CH alkyl), 1688 (CO, amide), 1639 (C=N), 1575 (C=C); ^1^H NMR (DMSO-*d6*, ppm) *δ* 1.18, 1.28 (2s, 6H, 2CH_3_), 7.01–7.96 (m, 11H, phenyl, quinoline), 8.26 (s, 1H, pyridine), 11.50, 11.77 (2brs, 2NH, D_2_O exchangeable); ^13^C NMR (DMSO-*d*_6_) *δ* 17.9 (2C, 2CH_3_), 115.8 (1C, CH, pyridine), 116.1, 117.6, 118.4, 119.5, 124.7, 126.5, 127.2, 127.8, 128.1, 129.5, 130.1, 136.2, 138.6, 144.4, 150.2, 153.8, 154.5, 155.7, 158.2, 160.2, 163.4 (23C, Ar-C, 23 sp^2^ carbon with four symmetric carbon atoms), 164.8 (1C, one carbonyl group), 175.1 (1C, C=S); and MS (70 ev, %): *m*/*z* = 520 (M^+^, 95%). Anal. Calc. for C_28_H_20_N_6_OS_2_ (520.63): C, 64.60 (64.52); H, 3.87 (3.83); N, 16.14 (16.20).

### 4.13. Synthesis of 2-(1-(Hydroxyimino)ethyl)-3-methyl-8-phenyl-6-(quinolin-2-yl)-5H-pyrido[2,3-d]thiazolo[3,2-a] Pyrimidin-5-one (**8**)

A mixture of 5 (4.62 g, 10 mmol) and hydroxylamine hydrochloride (0.70 mL, 10 mmol) in dioxane (30 mL) and a catalytic amount of piperidine was added. The reaction mixture was stirred under reflux for 17 h, cooled to room temperature, and poured into water (100 mL). The deposited precipitate was filtered off, dried, and crystallized from dioxane with a yield of (76%), m. p. > 350 °C (melted); IR (*ν*, cm^−1^) KBr: 3520 (brs, OH), 3066 (CH aryl), 2955 (CH alkyl), 1692, (CO, amide), 1644 (C=N); 1573 (C=C); ^1^H NMR (DMSO-*d6*, ppm) *δ* 2.18 (s, 3H, CH_3_), 2.20 (s, 3H, CH_3_), 6.88–7.91 (m, 11H, phenyl, quinoline), 8.07 (s, 1H, pyridine), 11.95 (br., OH, D_2_O exchangeable); ^13^C NMR (DMSO-*d*_6_) *δ* 17.10, 23.30 (2C, 2CH_3_), 114.4 (1C, CH, pyridine), 115.1, 116.9, 117.5, 120.8, 123.5, 126.7, 127.1, 127.5, 128.1, 128.6, 129.5, 136.2, 138.9, 143.3, 145.5, 150.2, 154.1, 154.5, 157.4, 159.3, 162.2 (23C, Ar-C, 23 sp^2^ carbon with four symmetric carbon atoms), 166.70 (1C, carbonyl group); and MS (70 ev, %): *m*/*z* = 477 (M^+^, 94%). Anal. Calc. for C_27_H_19_N_5_O_2_S (477.54): C, 67.91 (67.96); H, 4.01 (4.07); N, 14.67 (14.55).

### 4.14. Synthesis of 2-(3-(4-Substituted-phenyl) Acryloyl)-3-methyl-8-phenyl-6-(quinolin-2-yl)-5H-pyrido[2,3-d]thiazolo[3,2-a] Pyrimidin-5-one (**9a**–**c**)

General Procedure: A mixture of **5** (4.62 g, 10 mmol), aryl aldehyde (10 mmol) in ethanol (30 mL), and a catalytic amount of piperidine was heated and refluxed at 170–180 °C for 4–7 h. The product was solidified by cooling and adding methanol (50 mL). The precipitate formed was collected by filtration and crystallized from the appropriate solvent.

### 4.15. Synthesis of 2-Cinnamoyl-3-methyl-8-phenyl-6-(quinolin-2-yl)-5H-pyrido[2,3-d]thiazolo[3,2-a] Pyrimidin-5-one (**9a**)

The compound was obtained from benzaldehyde (1.06 g, 10 mmol) as yellow crystals, crystallized from dimethyl-formamide in 73% yield, m. p. > 350 °C (melted); IR (*ν*, cm^−1^) KBr: IR (*ν*, cm^−1^) KBr: 3062 (CH aryl), 2951 (CH alkyl), 1710 (CO, ketone), 1688 (CO, amide), 1637 (C=N); 1570 (C=C); ^1^H NMR (DMSO-*d6*, ppm) *δ* 3.23 (s, 3H, CH_3_), 5.47, 5.64 (2d, 2H, CH=CH, *J =* 10.60 Hz), 6.87–7.91 (m, 16H, two phenyl, quinoline), 8.06 (s, 1H, pyridine); ^13^C NMR (DMSO-*d*_6_) *δ* 18.90 (1C, CH_3_), 115.7 (1C, CH, pyridine), 116.9, 140.5 (2C, CH=CH), 117.5, 118.8, 119.1, 121.4, 124.5, 126.6, 126.9, 127.7, 127.8, 128.2, 128.5, 128.8, 129.3, 130.1, 134.7, 137.4, 139.2, 144.5, 150.6, 154.3, 155.1, 155.8, 156.5, 158.7 (28C, Ar-C, 28 sp^2^ carbon with four symmetric carbon atoms), 167.10, 182.5 (2C, two carbonyl groups); and MS (70 ev, %): *m*/*z* = 550 (M^+^, 97%). Anal. Calc. for C_34_H_22_N_4_O_2_S (550.64): C, 74.16 (74.23); H, 4.03 (4.10); N, 10.18 (10.11).

### 4.16. Synthesis of 2-(3-(4-Chlorophenyl) Acryloyl)-3-methyl-8-phenyl-6-(quinolin-2-yl)-5H-pyrido[2,3-d]thiazolo[3,2-a] Pyrimidin-5-one (**9b**)

The compound was obtained from 4-chlorobenzaldehyde (1.40 g, 10 mmol) as yellowish crystals crystallized from tetrahydrofuran, in 70% yield, m. p. > 350 °C (melted); IR (*ν*, cm^−1^) KBr: IR (*ν*, cm^−1^) KBr: 3060 (CH aryl), 2950 (CH alkyl), 1715 (CO, ketone), 1691 (CO, amide), 1633 (C=N); 1574 (C=C); ^1^H NMR (DMSO-*d6*, ppm) *δ* 2.91 (s, 3H, CH_3_), 5.60, 5.80 (2d, 2H, CH=CH, *J =* 10.65 Hz), 7.02–8.01 (m, 15H, two phenyl, quinoline), 8.25 (s, 1H, pyridine); ^13^C NMR (DMSO-*d*_6_) *δ* 19.2 (1C, CH_3_), 115.2 (1C, CH, pyridine), 117.8, 140.1 (2C, CH=CH), 118.2, 118.7, 119.5, 121.1, 125.3, 126.7, 127.1, 127.8, 128.1, 128.5, 129.1, 129.4, 130.2, 132.1, 134.5, 136.8, 139.3, 144.2, 150.4, 154.2, 155.3, 155.6, 156.4, 158.1 (28C, Ar-C, 28 sp^2^ carbon with four symmetric carbon atoms), 168.10, 185.30 (2C, two carbonyl groups); and MS (70 ev, %): *m*/*z* = 585 (M^+^, 98%). Anal. Calc. for C_34_H_21_ClN_4_O_2_S (585.08): C, 69.80 (69.75); H, 3.62 (3.70); N, 9.58 (9.63).

### 4.17. Synthesis of 2-(3-(4-Methoxyphenyl) Acryloyl)-3-methyl-8-phenyl-6-(quinolin-2-yl)-5H-pyrido[2,3-d]thiazolo[3,2-a] Pyrimidin-5-one (**9c**)

The compound was obtained from 4-methoxybenzaldehyde (1.36 g, 10 mmol) as brownish crystals, crystallized from n-hexane in 68% yield, m. p. > 350 °C (melted); IR (*ν*, cm^−1^) KBr: IR (*ν*, cm^−1^) KBr: 3057 (CH aryl), 2948 (CH alkyl), 1705 (CO, ketone), 1686 (CO, amide), 1630 (C=N); 1568 (C=C); ^1^H NMR (DMSO-*d6*, ppm) *δ* 2.95 (s, 3H, CH_3_), 3.90 (s, 3H, OCH_3_), 5.65, 5.85 (2d, 2H, CH=CH, *J =* 10.70 Hz), 7.05–8.07 (m, 15H, two phenyl, quinoline), 8.20 (s, 1H, pyridine); ^13^C NMR (DMSO-*d*_6_) *δ* 20.1 (1C, CH_3_), 52.5 (1C, OCH_3_), 114.7 (1C, CH, pyridine), 116.8, 140.6 (2C, CH=CH), 117.1, 118.3, 119.4, 120.3, 123.5, 124.2, 126.9, 127.2, 127.7, 127.9, 128.2, 129.3, 129.8, 130.4, 137.3, 139.1, 143.8, 150.1, 154.3, 155.1, 155.8, 156.5, 158.7, 160.1 (28C, Ar-C, 28 sp^2^ carbon with four symmetric carbon atoms), 166.90, 184.70 (2C, two carbonyl groups); and MS (70 ev, %): *m*/*z* = 580 (M^+^, 96%). Anal. Calc. for C_35_H_24_N_4_O_3_S (580.66): C, 72.40 (72.47); H, 4.17 (4.12); N, 9.65 (9.60).

### 4.18. Synthesis of 2-(1-(3-Methyl-5-oxo-8-phenyl-6-(quinolin-2-yl)-5H-pyrido[2,3-d]thiazolo[3,2-a] Pyrimidin-2-yl)ethylidene) Hydrazine-1-carbothioamide (**10**)

A mixture of **5** (4.62 g, 10 mmol) and thiosemicarbazide (0.91 g, 10 mmol) in dioxane (40 mL) and a catalytic amount of piperidine was added. The reaction mixture was stirred under reflux for 15 h, allowed to cool to room temperature, and poured into water (100 mL). The deposited precipitate was filtered off, dried, and crystallized from tetrahydrofuran. The compound was obtained and was pale-yellow in 81% yield, m. p. > 350 °C (melted); IR (*ν*, cm^−1^) KBr: 3350–3410 (brs, NH, NH_2_), 3077 (CH aryl), 2952 (CH alkyl), 1681, (CO, amide), 1629 (C=N); 1573 (C=C); ^1^H NMR (DMSO-*d6*, ppm) *δ* 1.10 (s, 3H, CH_3_), 2.23 (s, 3H, CH_3_), 4.90 (br., 2H, NH_2_, D_2_O exchangeable), 7.10–7.99 (m, 11H, phenyl, quinoline), 8.00 (s, 1H, pyridine), 9.40 (br., NH, D_2_O exchangeable); ^13^C NMR (DMSO-*d*_6_) *δ* 14.6, 21.1 (2C, 2CH_3_), 75.5 (1C, thiazole), 115.6 (1C, CH, pyridine), 153.7 (1C, -C=N-NH), 115.9, 118.2, 121.1, 124.6, 124.8, 128.1, 129.6, 130.3, 133.3, 134.5, 136.9, 140.4, 142.3, 152.8, 153.6, 159.9, 160.1, 163.3, 167.8 (21C, Ar-C, 21 sp^2^ carbon with four symmetric carbon atoms), 168.10 (1C, carbonyl group), 171.95 (1C, C=S); and MS (70 ev, %): *m*/*z* = 535 (M^+^, 100%). Anal. Calc. for C_28_H_21_N_7_OS_2_ (535.64): C, 62.79 (62.85); H, 3.95 (3.88); N, 18.30 (18.24).

### 4.19. Synthesis of 3-Methyl-2-(1-(5-(2-oxopropyl)-3-thioxo-2,3-dihydro-1H-1,2,4-triazol-1-yl)ethyl)-8-phenyl-6-(quinolin-2-yl)-5H-pyrido[2,3-d]thiazolo[3,2-a] Pyrimidin-5-one (**11**)

A solution of compound **10** (5.35 g, 10 mmol) and ethyl acetoacetate (1.30 g, 10 mmol) in a sodium ethoxide solution (prepared by dissolving 0.23 g of sodium metal in 35 mL ethanol) was heated under reflux with stirring for 9 h. The reaction mixture was allowed to cool and poured into cold water (100 mL). Acetic acid neutralized it, with it precipitating as a solid, and then filtering off and crystallizing from methanol as a yellowish crystal in 80% yield, m. p. > 350 °C (melted); IR (*ν*, cm^−1^) KBr: 3250 (brs, NH), 3084 (CH aryl), 2960 (CH alkyl), 1712 (CO, ketone), 1693 (CO, amide), 1632 (C=N); 1580 (C=C); ^1^H NMR (DMSO-*d6*, ppm) *δ* 1.80 (d, 3H, CH_3_), 1.90 (s, 3H, CH_3_), 2.83 (s, 3H, CH_3_), 3.65 (s, 2H, CH_2_), 3.71 (q, 1H, CH), 7.16–7.99 (m, 11H, phenyl, quinoline), 8.13 (s, 1H, pyridine), 8.76 (br., NH, D_2_O exchangeable); ^13^C NMR (DMSO-*d*_6_) *δ* 15.5, 17.1, 28.9 (3C, 3CH_3_), 39.7 (1C, CH_2_), 57.5 (1C, CH), 100.8 (1C, thiazole), 115.3 (1C, CH, pyridine), 116.6, 117.9, 120.2, 123.5, 126.8, 127.4, 127.8, 128.2, 129.3, 130.1, 133.4, 137.2, 139.4, 144.7, 150.1, 154.3, 154.6, 156.5, 158.8, 163.1 (22C, Ar-C, 22 sp^2^ carbon with four symmetric carbon atoms), 167.20, 171.5 (2C, carbonyl group), 185.30 (1C, C=S); and MS (70 ev, %): *m*/*z* = 603 (M^+^, 100%). Anal. Calc. for C_32_H_25_N_7_O_2_S_2_ (603.72): C, 63.66 (63.60); H, 4.17 (4.25); N, 16.24 (16.30).

### 4.20. Synthesis of 3-Methyl-2-(1-(7-methyl-3-thioxo-2,3-dihydro-[1,2,4]triazolo[4,3-c]pyrimidin-1(5H)-yl)ethyl)-8-phenyl-6-(quinolin-2-yl)-5H-pyrido[2,3-d]thiazolo[3,2-a] Pyrimidin-5-one (**12**)

A mixture of compound **11** (6.03 g, 10 mmol) and formamide (15 mL) was stirred under reflux in dimethylformamide (45 mL) for 8 h under control (TLC). The reaction mixture was allowed to cool to room temperature, poured into water (100 mL), and neutralized by an ammonia solution. The precipitate was collected by filtration, washed with water–ethanol, dried, and crystallized from ethanol as a yellow crystal in 78% yield, m. p. > 350 °C (melted); IR (*ν*, cm^−1^) KBr: 3310 (brs, NH), 3088 (CH aryl), 2970 (CH alkyl), 1687, (CO, amide), 1635 (C=N); 1575 (C=C); ^1^H NMR (DMSO-*d6*, ppm) *δ* 1.98 (d, 3H, CH_3_), 2.29 (s, 3H, CH_3_), 2.32 (s, 3H, CH_3_), 3.82 (q, 1H, CH), 4.32 (s, 2H, CH_2_, pyrimidine), 6.90–7.99 (m, 11H, phenyl, quinoline), 8.02 (s, 1H, pyridine), 8.11 (s, 1H, pyrimidine), 8.74 (br., NH, D_2_O exchangeable); ^13^C NMR (DMSO-*d*_6_) *δ* 15.6, 17.3, 20.5 (3C, 3CH_3_), 60.7 (1C, CH), 70.1 (1C, CH, pyrimidine), 78.5 (1C, CH_2_, pyrimidine), 101.30 (1C, thiazole), 115.8 (1C, CH, pyridine), 117.2, 118.4, 120.5, 124.1, 126.9, 127.1, 127.5, 128.4, 129.1, 130.5, 132.8, 137.5, 139.1, 144.2, 150.6, 154.1, 154.7, 156.4, 157.1, 158.5, 164.2 (23C, Ar-C, 23 sp^2^ carbon with four symmetric carbon atoms), 166.70, (1C, carbonyl group), 180.50 (1C, C=S); and MS (70 ev, %): *m*/*z* = 614 (M^+^, 98%). Anal. Calc. for C_33_H_26_N_8_OS_2_ (614.75): C, 64.48 (64.55); H, 4.26 (4.20); N, 18.23 (18.30).

### 4.21. Synthesis of 3-Methyl-2-(1-(7-methyl-5-(pyridin-4-ylmethylene)-3-thioxo-2,3-dihydro-[1,2,4]triazolo[4,3-c]pyrimidin-1(5H)-yl)ethyl)-8-phenyl-6-(quinolin-2-yl)-5H-pyrido[2,3-d]thiazolo[3,2-a] Pyrimidin-5-one (**13**)

Method A: A mixture of 6.14 g (10 mmol) of compound (**12**) and 1.07 g (10 mmol) of isonicotinaldehyde in 35 mL of dioxane with 0.5 mL of piperidine as a catalyst was stirred and heated under reflux for 8 h with TLC monitoring. After cooling, the precipitate formed was filtered, dried, and recrystallized from dimethylformamide.

Method B: A solution containing **12** (6.14 g, 10 mmol) and isonicotinaldehyde (1.07 g, 10 mmol) with anhydrous sodium acetate (1.64 g, 20 mmol) was heated and stirred under reflux in 40 mL of glacial acetic acid and 20 mL of acetic anhydride for 7 h while monitoring with TLC. The reaction mixture was then cooled to room temperature and poured into 100 mL of cold water. The resulting precipitate was filtered and crystallized from dimethylformamide, as white crystal with a 75% yield, m. p. > 350 °C (melted); IR (*ν*, cm^−1^) KBr: 3322 (brs, NH), 3091 (CH aryl), 2983 (CH alkyl), 1693, (CO, amide), 1631 (C=N); 1577 (C=C); ^1^H NMR (DMSO-*d6*, ppm) *δ* 1.27 (d, 3H, CH_3_), 1.63 (s, 3H, CH_3_), 2.05 (s, 3H, CH_3_), 3.88 (q, 1H, CH), 6.68 (s, 1H, CH, methine proton), 7.01–7.78 (m, 11H, phenyl, quinoline), 7.80–7.88 (d, 2H, *J* = 7.90 Hz, pyridine), 7.90–7.98 (d, 2H, *J* = 7.95 Hz, pyridine), 8.08 (s, 1H, pyridine), 8.10 (s, 1H, pyrimidine), 10.68 (br., NH, D_2_O exchangeable); ^13^C NMR (DMSO-*d*_6_) *δ* 15.7, 17.4, 21.1 (3C, 3CH_3_), 61.2 (1C, CH), 70.8 (1C, CH, pyrimidine), 95.1 (1C, CH, methine proton), 101.70 (1C, thiazole), 116.3 (1C, CH, pyridine), 117.6, 118.9, 120.8, 123.9, 124.5, 127.1, 127.4, 127.7, 128.1, 129.4, 130.2, 133.5, 137.1, 139.3, 142.2, 143.5, 144.4, 145.7, 148.9, 150.5, 154.3, 154.5, 156.1, 158.4, 166.2 (29C, Ar-C, 29 sp^2^ carbon with four symmetric carbon atoms), 167.50, (1C, carbonyl group), 181.60 (1C, C=S); and MS (70 ev, %): *m*/*z* = 703 (M^+^, 97%). Anal. Calc. for C_39_H_29_N_9_OS_2_ (703.84): C, 66.55 (66.62); H, 4.15 (4.10); N, 17.91 (17.84).

### 4.22. Synthesis of 4-Methyl-2-(1-(3-methyl-5-oxo-8-phenyl-6-(quinolin-2-yl)-5H-pyrido[2,3-d]thiazolo[3,2-a] Pyrimidin-2-yl)ethyl)-6-(pyridin-4-yl)-2H-9-thia-1,2,2a1,5-tetraazabenzo[cd]azulen-7(8H)-one (**14**)

The following reaction was carried out: a mixture of 7.03 g, 0.01 mol of compound (**13**); 1.70 mL, 0.01 mol of ethyl bromoacetate; and 2.07 g, 0.015 mol of anhydrous potassium carbonate in 40 mL of dry acetone was refluxed for 12 h under control (TLC). Upon cooling to room temperature, 50 mL of water was added. The separated solid was filtered, dried, and then crystallized from dioxane, sa yellowish crystal in 72% yield, m. p. > 350 °C (melted); IR (*ν*, cm^−1^) KBr: 3095 (CH aryl), 2988 (CH alkyl), 1718, (CO, ketone), 1695, (CO, amide), 1636 (C=N); 1585 (C=C); ^1^H NMR (DMSO-*d6*, ppm) *δ* 1.10 (d, 3H, CH_3_), 2.24 (s, 3H, CH_3_), 3.75 (s, 3H, CH_3_), 4.01 (q, 1H, CH), 4.85 (s, 2H, CH_2_, thiazepine ring), 6.88–7.80 (m, 11H, phenyl, quinoline), 7.82–7.90 (d, 2H, *J* = 7.93 Hz, pyridine), 7.91–7.99 (d, 2H, *J* = 7.97 Hz, pyridine), 8.03 (s, 1H, pyrimidine), 8.10 (s, 1H, pyridine); ^13^C NMR (CDCl_3,_ ppm) *δ* 14.28, 24.47, 26.09 (3C, 3CH_3_), 37.00 (1C, CH_2_, thiazepine ring), 64.73 (1C, CH), 70.00 (1C, CH, pyrimidine), 112.95 (1C, thiazole), 113.25 (1C, CH, pyridine), 113.6, 113.8, 114.1, 114.3, 116.8, 121.9, 122.0, 122.1, 122.2, 125.7, 126.0, 128.9, 129.1, 129.6, 129.7, 130.2, 130.3, 137.7, 145.5, 145.6, 146.4, 146.9, 147.0, 155.8, 161.8, 161.9, 164.2 (31C, Ar-C, 31 sp^2^ carbon with four symmetric carbon atoms), 164.4, 168.0 (2C, two carbonyl groups); and MS (70 ev, %): *m*/*z* = 743 (M^+^, 95%). Anal. Calc. for C_41_H_29_N_9_O_2_S_2_ (743.86): C, 66.20 (66.27); H, 3.93 (3.88); N, 16.95 (16.90).

### 4.23. Biological Screening (Materials and Methods, In Vitro)

The newly prepared compounds were tested for their antimicrobial activity in vitro against the following microorganisms: Gram-positive bacteria *Streptococcus pyogenes* (ATCC^®^ 19615^™^) and *Staphylococcus aureus* (ATCC^®^ 6538^™^); Gram-negative bacteria *Klebsiella pneumoniae* (ATCC^®^ 10031^™^) and *Escherichia coli* (ATCC^®^ 25922^™^); and the fungi *Candida albicans* (ATCC^®^ 10231^™^), *Curvularia lunata*, *Alternaria alternate*, and *Aspergillus niger* (ATCC^®^ 16888^™^). The newly synthesized compounds were dissolved in dimethyl sulfoxide (DMSO), and their antimicrobial activity was tested using agar disk diffusion. Cefotaxime sodium and nystatin [32,76,77,78,79,80] were used as standard drugs for the antibacterial and antifungal assays, respectively. A solution of 100 μg mL^−1^ of the tested compound was used, and microplate wells 1 cm in diameter were employed. Zones of inhibition were measured with calipers or automated scanners and compared with those of the standards. Cefotaxime sodium (0.15 μmol mL^−1^) and nystatin (0.037 μmol mL^−1^) were used as the standard antibacterial and antifungal drugs. Compound-impregnated disks were placed on an agar plate containing a standard suspension of microorganisms, and the plate was then incubated for 24 h at 37 °C. To determine the minimum inhibitory concentration (MIC) by serial plate dilution [32,76,77,78,79,80], 5 mg of each tested compound was dissolved in 1 mL of DMSO separately to prepare stock solutions. Serial dilutions were prepared from each stock solution, and the plates were incubated at 37 °C for 24 h. The MIC is defined as the lowest concentration (μmol mL^−1^) of the tested compound, resulting in no visible growth on the plates. DMSO was used as the solvent control to ensure that the solvent did not affect bacterial growth. The results are presented in Table 1 and Table 2.

#### 4.23.1. Ethical Approval and Consent to Participate

This study did not involve any humans or animals. However, all procedures were conducted with the approval of the Medical Research Ethics Committee of the National Research Centre, Department of Chemistry of Natural and Microbial Products in Giza, Egypt 12622.

#### 4.23.2. Human and Animal Rights

This study did not use human or animal subjects. It was conducted in vitro according to ethical standards.

#### 4.23.3. Chemicals and Drugs

The following types of bacteria and fungi were obtained from the Department of Chemistry of Natural and Microbial Products at the National Research Centre in Giza, Egypt: Gram-positive bacteria *Streptococcus pyogenes* and *Staphylococcus aureus*; Gram-negative bacteria *Klebsiella pneumoniae* and *Escherichia coli*; and fungi *Candida albicans*, *Curvularia lunata*, *Alternaria alternate*, and *Aspergillus niger*. Additionally, cefotaxime sodium, nystatin, and DMSO were purchased from Sigma-Aldrich.

## 5. Conclusions

Several new compounds containing thiazole, pyrazole, hydrazine, 1,2,4-triazole, and 1, 3-thiazepinone moieties linked to quinoline-pyrido[2,3-*d*] pyrimidinone (**2**–**14**) and its derivatives were synthesized using recent methods and high techniques, resulting in excellent yields. The antimicrobial activities of these compounds against various bacteria and fungi were investigated in vitro. The new compounds (**2**–**14**) were synthesized using multiple reagents, and their high purity was confirmed through physical and analytical data and spectroscopy analysis. After being screened for antimicrobial activity against selected strains of bacteria and fungi, compounds 1,3-thiazepinone, 1,2,4-triazolopyrimidine linked to quinoline-pyrido[2,3-*d*]thiazolo[3,2-*a*] pyrimidine (**10**–**14**), and derivatives showed excellent activity, and this is likely due to the presence of thiazole, 1,2,4-triazole, 1,3-thiazepinone, phenyl, pyridine, pyrimidine, and quinoline rings, as well as various functional groups like thioxo, carbonyl, NH, and methyl moiety. In this manuscript, we evaluated the antimicrobial effectiveness of various extracts against pathogenic microorganisms. Subsequently, we used molecular docking to study the interactions between promising compounds and antimicrobial target proteins. Compounds **10**, **11**, **12**, **13**, and **14** demonstrated vital binding energy, effectively interacting with the active sites of antimicrobial protein receptors. These interactions suggest the potential for enzyme inhibition and significant antimicrobial benefits. The results underscore the compounds’ potential in current drug development initiatives, emphasizing their stability and suitability for further investigation in drug development processes.

Thus, these compounds (**10**–**14**) showed better activity than the standard drugs cefotaxime sodium and nystatin. We expect researchers to develop these compounds into drugs with antimicrobial properties.

## Figures and Tables

**Table 1:** A minimum inhibitory concentration of the compounds against bacteria.

MIC (µmol mL^−1^)				
Compounds	Microorganisms			
	Gram-Positive Bacteria		Gram-Negative Bacteria	
	*Streptococcus pyogenes*	*Staphylococcus aureus*	*Klebsiella pneumoniae*	*E. coli*
**1**	16	17	18	19
**2a**	15	16	17	18
**2b**	14	15	16	17
**3a**	11	12	12	13
**3b**	10	11	10	11
**4**	12	13	14	15
**5**	9	10	9	10
**6**	7	7	6	7
**7**	6	7	6	7
**8**	8	9	8	8
**9a**	5	6	6	5
**9b**	3	4	3	4
**9c**	4	5	4	5
**10**	2	3	2	3
**11**	1	2	2	2
**12**	1	2	1	2
**13**	1	2	1	1
**14**	1	1	1	1
Cefotaxime sodium	1	2	1	1
Negative control	NI	NI	NI	NI

DMSO was used as the negative control and solvent for test compounds and the reference drug.

**Table 2:** A minimum inhibitory concentration of the compounds against fungi.

MIC (µmol mL^−1^)				
Compounds	Microorganisms			
	*Candida albicans*	*Curvularia lunata*	*Alternaria alternata*	*Aspergillus niger*
**1**	20	21	22	23
**2a**	18	19	20	21
**2b**	17	18	19	20
**3a**	14	15	16	17
**3b**	12	13	14	15
**4**	15	16	17	18
**5**	10	11	12	13
**6**	8	9	10	11
**7**	7	8	9	10
**8**	9	10	11	12
**9a**	6	7	7	8
**9b**	4	5	6	6
**9c**	5	6	7	7
**10**	3	4	4	5
**11**	2	2	3	4
**12**	1	2	2	3
**13**	1	2	1	2
**14**	1	1	1	2
Nystatin	1	1	2	3
Negative control	NI	NI	NI	NI

DMSO was used as the negative control and solvent for test compounds and the reference drug.

**Table 3:** Targets of antimicrobial proteins, PDB IDs, active site coordinates, and reference.

Microorganism	Protein Targets	PDB ID	Resolutions	Active Sit Coordinates:			References [77,78,79,80,83,84,85,86,90,91]
				X	Y	Z	
*S. aureus*	Dihydropteroate Synthetase	1M17	2.60 Å	19.96	5.08	20.12	cefotaxime
*E. coli*	GyrB24	7P2M	1.16 Å	72.07	36.33	11.54	cefotaxime
*K. pneumoniae*	KPC-2 carbapenemase	3ERT	1.90 Å	27.52	63.35	−2.29	cefotaxime
*S. pyogenes*	Sortase A	8T8G	1.50 Å	72.07	36.33	11.54	cefotaxime
*C. albicans*	Sterol 14-alpha demethylase	5TZ1	2.00 Å	19.96	5.08	20.12	nystatin
*Aspergillus niger*	fdc1 protein	4ZA5	1.10 Å	27.52	63.35	−2.29	nystatin
*A. alternata*	Crystal structure of AaTPS	7P2M	1.16 Å	72.07	36.33	11.54	nystatin

**Table 4:** Molecular interactions with amino acids of dihydropteroate synthase of *S. aureus* (PDB: ID 1AD4).

	Protein	Ligand	Hydrophilic Interactions	Hydrophobic Contacts			No. of H-Bonds	No. of Total Bonds	Affinity kcal mol^−1^
			Residue (H-Bond)	Length	Residue (Bond Type)	Length			
1	Dihydropteroate synthase of *S. aureus*	**10**	Asn11 (H-bond) His241 (H-bond) Asp84 (H-bond)	4.55 2.60 2.91	Ile9, (pi–alkyl) Asp84, (pi–cation) His241, (pi–cation) Pro216, (pi–alkyl) Phe172, (pi–sulfur) His241, (pi–alkyl)	4.44 5.10 5.34 5.11 5.30 5.34	3	12	−8.90
2		**11**	Arg239 (H-bond) Asp84 (H-bond) Asn103 (H-bond)	1.92 2.38 2.87	Arg52, (pi–alkyl) Phe172, (pi–alkyl) Phe172, (pi–pi shaped) Arg239, (pi–cation)	4.82 4.88 4.98 3.54	3	7	−8.80
3		**12**	-		Arg52, (pi–alkyl) His241, (pi–pi shaped)	3.91 5.30	2	10	−8.70
4		**13**	Met128 (H-bond)	2.57	Ala199, (pi–alkyl) Arg239, (pi–cation) Phe172, (pi–alkyl) Met128, (pi–alkyl) Val126, (pi–alkyl) Met128, (pi–sulfur) Phe172, (pi–pi shaped)	3.30 3.69 4.81 5.06 4.82 3.59 4.81	1	8	−8.40
5		**14**	Arg52 (H-bond)	2.54	Ile9, (pi–alkyl) His55, (pi–alkyl) Arg52, (pi–alkyl) Arg239, (pi–cation) His241, (pi–pi shaped) Arg202, (CH-bond)	4.99 3.67 3.34 3.31 4.74 3.18	1	11	−8.50
6		cefotaxime	Gly54 (H-bond) Tyr56 (H-bond)	2.73 2.91	Ala58, (halogen) Ile52, (pi–alkyl) Asp65, (pi–cation) Arg61, (pi–cation) Arg61, (pi–alkyl)	3.12 4.42 3.49 4.15 5.01	2	9	−6.10

**Table 5:** Molecular interactions of ligands with amino acids of DNA gyrase of *E. coli* (PDB: ID 7P2M).

No.	Protein	Ligand	Hydrophilic Interactions		Hydrophobic Contacts		No. of H-Bonds	No. of Total Bonds	Affinity kcal mol^−1^
			Residue (H-Bond)	Length	Residue (Bond Type)	Length			
1	DNA gyrase of *E.coli* (PDB: ID 7P2M)	**10**	Leu98, (H-bond) Leu98, (H-bond)	2.78 2.68	Ile94, (pi–sigma) Ile94, (pi–sigma) Ile78, (pi–sigma) Val120, (alkyl) Arg76, (pi–cation)	3.68 3.62 3.67 5.42 3.66	2	8	−8.90
2		**11**	-	-	Ile94, (pi–sigma) Ile94, (pi–sigma) Ile78, (pi–sigma) Val120, (alkyl) Arg76, (pi–cation)	3.82 3.67 3.65 3.65 3.81	0	5	−8.40
3		**12**	-	-	Ile94, (pi–sigma) Ile94, (pi–sigma) Ile78, (pi–sigma) Val120, (alkyl) Arg76, (pi–cation) Leu98, (alkyl) Asn46, (pi–pi T-shaped)	3.68 3.47 3.71 5.01 3.82 4.86 5.05	0	8	−8.10
4		**13**	-	-	Thr165, (pi–sigma) Asp49, (pi–cation) Leu98, (C-H bond) Gly77, (pi–pi T-shaped) Ile94, (alkyl) Pro79, (alkyl Ile78, (alkyl) Ala47, (alkyl) Val43, (alkyl) Val167, (alkyl) Val120, (alkyl)	3.71 3.35 4.13 5.16 5.12 4.28 4.23 5.14 5.15 4.82 3.80	0	11	−8.10
		**14**	-	-	Ile94, (pi–sigma) Ile94, (pi–sigma) Ile78, (alkyl) Ile94, (alkyl)	3.59 3.00 5.31 4.80	0	5	−8.10
5		cefotaxime	Ala47, (H-bond) Glu50, (H-bond) Ser121, (H-bond) Ile94, (H-bond)	2.93 2.65 2.83 2.01	Val120, (alkyl) Ile78, (alkyl) Asp73, (C-H bond)	5.09 4.25 3.40	4	7	−7.00

**Table 6:** Molecular interactions of ligands with Sortase A in *S. pyogenes*.

	Protein	Ligand	Hydrophilic Interactions	Hydrophobic Contacts			No. of H-Bonds	No. of Total Bonds	Affinity kcal mol^−1^
			Residue (H-Bond)	Length	Residue (Bond Type)	Length			
1	Sortase A in S. pyogenes (PDB: ID 8T8G)	**10**	-	-	Val206, (pi–sigma) Leu113, (pi–sigma) Arg216, (pi–cation) Arg216, (pi–cation) Arg216, (pi–alkyl) Ala208, (pi–alkyl) Met125, (pi–sulfur)	3.98 3.38 4.78 4.28 5.05 4.01 5.55	0	7	−8.20
2		**11**	-	-	Val206, (pi–sigma) Leu113, (pi–sigma) Arg216, (pi–cation) Arg216, (pi–cation) Arg216, (pi–alkyl) Ala208, (pi–alkyl) Met125, (pi–sulfur)	3.94 5.07 2.57	0	7	−7.90
		**12**	-	-	Val206, (pi–alkyl) Val206, (pi–alkyl) Ile194, (pi–alkyl) Val193, (pi–alkyl) Val191, (pi–sigma) Arg216, (pi–alkyl) Ala208, (pi–alkyl)	4.86 4.96 5.39 4.31 3.78 4.78 4.71		7	−7.80
		**13**	-	-	Pro188, (pi–alkyl) Leu113, (pi–alkyl) Val206, (pi–alkyl) Val206, (pi–alkyl) Lys126, (pi–alkyl) Ile194, (pi–alkyl) His142, (pi–pi T-shaped) Ile194, (pi–sigma)	4.60 5.16 5.25 4.75 5.23 3.97 3.97 3.28	0	11	−7.90
		**14**			Lys126, (pi–alkyl) Lys126, (pi–alkyl) Val206, (pi–alkyl) Leu11, (pi–alkyl) Ile218, (pi–alkyl) Val206, (pi–alkyl)	3.99 4.87 4.51 5.16 3.81 3.81	0	7	−8.00
3		cefotaxime	Gln246, (H-bond) Gln152, (H-bond). Gln152, (H-bond)	2.12 2.69 2.39	Leu103, (C-H bond) Gln152, (C-H bond)	3.67 3.51	3	5	−7.30

**Table 7:** Molecular interactions of ligands with amino acids of KPC-2 carbapenemase of *K. pneumoniae* (PDB: ID 2OV5).

No.	Protein	Ligand	Hydrophilic Interactions		Hydrophobic Contacts		No. of H-Bonds	No. of Total Bonds	Affinity kcal mol^−1^
			Residue (H-Bond)	Length	Residue (Bond Type)	Length			
1	KPC-2 carbapenemase of *K. pneumoniae* (PDB: ID 2OV5)	**10**	Glu166, (H-bond) Asn132, (H-bond) Thr237, (H-bond) Glu276, (H-bond)	2.39 2.96 2.00 3.05	Thr215, (pi–lone pair) Trp105, (pi–sigma) His219, (pi–cation) Glu276, (pi–cation)	2.86 3.44 3.00 4.86	4	8	−8.00
2		**11**	Ser130, (H-bond) Asn132, (H-bond) Thr237, (H-bond) Ser70, (H-bond)	2.69 2.03 2.73 2.08	His219, (pi–cation) His274, (pi–sigma) Thr216, (pi–sigma) His219, (pi–cation)	3.43 5.45 3.70 3.25	4	8	−8.40
		**12**	Asn132, (H-bond)	2.96	Trp105, (pi–sigma) Trp105, (alkyl) Trp105, (alkyl) Arg220, (alkyl) Glu276, (pi–cation) Glu276, (pi–cation) Glu166, (C-H bond)	3.74 5.08 4.46 4.03 4.73 2.42 3.76	1	8	−7.90
		**13**	Leu167, (H-bond)	2.88	Trp105, (pi–pi T-shaped) Trp105, (pi–pi T-shaped) Trp105, (alkyl) Trp105, (alkyl) Asn132, (C-H bond)	5.35 3.95 4.15 4.90 3.07	1	8	−7.80
		**14**	Thr216, (H-bond)	6.11	Trp105, (pi–pi T-shaped) Trp105, (pi–pi T-shaped) Leu167, (alkyl) His219, (pi–cation) Thr235, (pi–lone pair)	4.91 4.87 5.06 3.66 2.91	1	8	−8.10
6		cefotaxime	Ser130, (H-bond) Ser70, (H-bond) Asn132, (H-bond) Lys73, (H-bond) Thr237, (H-bond) Thr235, (H-bond) Thr215, (H-bond) Ser70, (H-bond)	2.12 2.63 2.48 2.90 2.58 2.10 2.48 2.63	Trp105, (pi–pi T-shaped) Trp105, (pi–sigma) Glu276, (pi–cation) Trp105, (pi–sulfur) Trp105, (pi–sulfur) His219, (C-H bond)	5.70 3.79 4.50 3.79 3.70 3.44	8	14	−8.40

**Table 8:** Molecular interactions of ligands with amino acids of sterol 14-alpha demethylase of *C. albicans* (PDB: ID 5TZ1).

	Protein	Ligand	Hydrophilic Interactions	Hydrophobic Contacts			No. of H-Bonds	No. of Total Bonds	Affinity kcal mol^−1^
			Residue (H-Bond)	Length	Residue (Bond Type)	Length			
1	sterol 14-alpha demethylase of *C. albicans* (PDB: ID 5TZ1)	**10**	Cys470, (H-bond)	2.12	Ile131, (pi–alkyl) Leu139, (pi–alkyl) Lys143, (pi–alkyl) Ile471, (pi–alkyl) Cys470, (pi–alkyl) Gly472, (pi–sigma) Leu300, (pi–sigma) Ile304, (pi–sigma) Phe463, (sulfur)	4.69 5.44 4.11 5.34 4.43 3.71 3.93 5.49	1	10	−10.20
2		**11**	Tyr132, (H-bond) Phe463, (H-bond) Gly465, (H-bond)	3.01 2.75 2.10	Leu376, (pi–alkyl) Ala476, (pi–alkyl) Ile304, (pi–alkyl) Ile471, (pi–alkyl) Lys143, (pi–alkyl) Lys143, (pi–alkyl) Ile379 (pi–sigma) Ile304, (pi–sigma) Leu300 (pi–sigma) Arg469, (pi–pi T-shaped) His468, (pi–pi T-shaped)	4.57 5.48 5.00 5.20 4.65 4.87 3.58 3.14 4.30 451 4.18	3	14	−9.90
3		**12**	Cys470, (H-bond) Cys470, (H-bond)	2.51 2.89	Tyr132, (pi–alkyl) Ala114, (pi–alkyl) Lys143, (pi–alkyl) Ala146, (pi–alkyl) Cys470, (pi–alkyl) Ala476, (pi–sigma) Leu276, (pi–sigma) Leu150, (pi–sigma) Cys470, (pi–sulfur)	5.36 3.83 5.12 4.71 6.30 3.35 3.97 5.24 5.69	2	12	−9.00
4		**13**	Ser312, (H-bond)	2.88	Phe463, (pi–alkyl) Pro375, (pi–alkyl) Leu480, (pi–alkyl) Ala476, (pi–alkyl) Lys143, (pi–alkyl) Ile131, (pi–alkyl) Ile471 (pi–alkyl) Ile304, (pi–alkyl) Leu204 (pi–alkyl) Leu300, (pi–sigma) Ala476, (pi–sigma) Arg469, (pi–pi T-shaped) Thr311 (pi–pi T-shaped) Thr315, (carbon–H bond)	4.81 5.22 4.96 3.94 4.52 4.43 5.32 4.83 5.31 3.65 3.45 4.76 4.00 2.33	1	15	−8.80
5		**14**	Pro462, (H-bond)	3.01	Phe475, (pi–alkyl) Ala146, (pi–alkyl) Ala149, (pi–alkyl) Ile471, (pi–alkyl) Ala476, (pi–sigma) Leu150, (pi–sigma) Leu276, (pi–sigma) Ile304, (pi–sigma) Arg469, (pi–pi T-shaped) Phe105, (pi–sulfur) Ile471, (carbon–H bond)	4.97 4.51 4.59 4.58 2.88 3.32 3.66 3.82 3.91 5.61 3.02	1	12	−8.80
5		nystatin	Cys470, (H-bond) Thr311, (H-bond) Pro462, (H-bond)	1.49 2.66 3.01	Cys470, (pi–alkyl) Met374, (carbon–H bond) Phe463, (carbon–H bond) Leu280, (pi–alkyl) Leu301, (pi–alkyl)	4.70 3.20 2.64 3.92 5.12	3	8	−7.20

**Table 9:** Interactions of compounds with amino acids of fdc1of *A.niger* (PDB: ID 4ZA5).

No.	Protein	Compounds	Hydrophilic Interactions		Hydrophobic Contacts		No. of H-Bonds	No. of Total Bonds	Affinity kcal mol^−1^
			Residue	Length	Residue (Bond Type)	Length			
1	fdc1of *A. niger* (PDB: ID 5TZ1)	**10**	Ile171, (H-bond) Ile171, (H-bond) Met283, (H-bond) Cys316, (H-bond) Gly281, (H-bond)	2.91 1.59 2.21 2.17 2.60	Phe437, (pi–alkyl) Pro226, (pi–alkyl) Ile327, (pi–alkyl) Thr395, (pi–sigma) Lys391, (pi–cation) Ser170, (pi–cation) Ser224, (pi–cation) Ile171, (pi–sulfur)	5.49 4.14 5.36 3.47 4.57 4.66 4.76 3.30	5	13	−1170
2		**11**	Ile171, (H-bond) Ile171, (H-bond)	3.06 1.68	Phe437, (pi–alkyl) Ile327, (pi–alkyl) Pro226, (pi–alkyl) Arg173, (pi–alkyl) Ile327, (pi–sigma) Lys391, (pi–cation) Ser170, (pi–cation) Met283, (pi–sulfur) Ile171, (pi–sulfur)	4.92 5.21 4.41 4.93 3.61 4.52 4.80 5.79 3.24	2	12	−10.50
3		**12**	Ile171, (H-bond) Ile171, (H-bond)	3.06 1.68	Phe437, (pi–alkyl) Ile327, (pi–alkyl) Pro226, (pi–alkyl) Arg173, (pi–alkyl) Arg439, (pi–alkyl) Ile327, (pi–sigma) Lys391, (pi–cation) Ser170, (pi–cation) Met283, (pi–sulfur) Ser170, (pi–cation) Ile171, (pi–sulfur)	4.98 3.71 4.34 4.94 5.07 5.33 4.53 4.83 4.71 5.81 3.25	2	13	−10.10
4		**13**	Ile171, (H-bond)	2.70	Phe437, (pi–alkyl) Leu185, (pi–alkyl) Pro226, (pi–alkyl) Arg173, (pi–alkyl) Cys316, (pi–alkyl) Thr395, (pi–sigma) Ser224, (pi–cation) Phe280, (pi–sulfur)	5.41 7.79 3.82 4.15 5.20 3.63 4.78 4.21	1	10	−11.60
5		**14**	Thr153, (H-bond) Ile171, (H-bond)	2.58 2.08	Pro226, (pi–alkyl) Phe437, (pi–alkyl) Ile396, (pi–alkyl) Tyr150, (pi–alkyl) Phe280, (pi–alkyl Met283, (pi–alkyl) Leu185, (pi–alkyl Ile171, (pi–sulfur) Ile171, (pi–sigma) Phe280, (amid–pi stack)	5.09 5.31 5.47 5.94 4.10 4.86 5.29 2.79 3.70 5.33	2	12	−11.70
6		nystatin	Arg173, (H-bond) Cys316, (H-bond) Thr153, (H-bond)	2.11 2.49 2.09	Ile171, (pi–alkyl) Ala331, (carbon–H bond)	3.24 3.43	3	5	−8.58

**Table 10:** Molecular interactions of ligands with amino acids of AaTPS of *Alternaria alternata* (PDB: ID 6LCD).

	Protein	Ligand	Hydrophilic Interactions	Hydrophobic Contacts			No. of H-Bonds	No. of Total Bonds	Affinity kcal mol^−1^
			Residue (H-Bond)	Length	Residue (Bond Type)	Length			
1	AaTPS of *Alternaria alternata* (PDB: ID 6LCD)	**10**	Glu137, (H-bond)	2.75	Ile173, (pi–alkyl) Ile172, (pi–alkyl) Ala269, (pi–alkyl) Ile328, (pi–alkyl) Asp176, (pi–cation) Asp267, (pi–cation) Arg264, (pi–cation) Glu137, (pi–cation) Tyr326, (amid–pi stack) Phe146, (pi–sigma)	3.85 4.94 4.98 5.50 3.77 3.23 4.42 3.33 5.93 3.96	1	14	−10.20
2		**11**	Asp176, (H-bond) Asp267, (H-bond) Asp267, (H-bond) Val268, (H-bond) Asn307, (H-bond)	1.87 2.11 1.66 5.34 3.04	Phe149, (pi–alkyl) Ile133, (pi–alkyl) Ile328, (pi–alkyl) Phe149, (pi–alkyl) Leu273, (pi–alkyl) Phe146, (pi–alkyl) Tyr326, (amid–pi stack) Arg124, (pi–cation) Phe146, (pi–sulfur)	4.62 5.45 5.19 4.62 5.34 4.12 3.98 3.58 5.91	5	15	−9.90
3		**12**	Asn392, (H-bond)	2.67	Val303, (pi–alkyl) Ile328, (pi–alkyl) Leu273, (pi–alkyl) Ile172, (pi–alkyl) Ala269, (pi–alkyl) Asp176, (pi–cation) Phe149, (pi–sigma) Tyr326, (amid–pi stack) Asp267, (pi–cation)	4.64 5.09 5.05 3.85 3.55 3.31 3.95 4.61 4.82	1	10	−9.00
4		**13**	-	-	Ile328, (pi–alkyl) Val268, (pi–alkyl) His136, (pi–alkyl) His136, (pi–cation) Asp176, (pi–cation) Asp267, (pi–cation) Tyr326, (amid–pi stack)	5.12 5.12 5.27 4.71 4.85 3.87 4.90	0	6	−8.80
5		**14**	Glu137, (H-bond) Asp176, (H-bond) Asp176, (H-bond) Tyr326, (H-bond) Tyr326, (H-bond)	2.38 2.19 2.46 2.18 1.66	Val268, (pi–alkyl) Lys314, (pi–alkyl) Ile328, (pi–alkyl) Tyr326, (amid–pi stack)	5.47 4.62 4.95 4.32	5	10	−7.20
6		nystatin	Ile266, (H-bond) Val268, (H-bond)	2.27 2.69	His136, (pi–alkyl) His136, (pi–alkyl) Lys314, (C-H bonds)	4.95 4.21 2.31			−7.00

## Data Availability

All data are contained within the article.
